# Diagnostic and Prognostic Value of Hypoxia PET in Glioma: A Systematic Review and Meta-Analysis

**DOI:** 10.3390/cancers18121898

**Published:** 2026-06-10

**Authors:** Aly Muhammad Ladak, Seyed Ali Mirshahvalad, Adam Farag, Ur Metser, Claudia Ortega, Vanessa Murad, Patrick Veit-Haibach

**Affiliations:** 1 University Medical Imaging Toronto, Joint Department of Medical Imaging, University Health Network, Sinai Health System & Women’s College Hospital, Toronto, ON M5G 2N2, Canada; 2 Department of Medical Imaging, Health Sciences North, Northern Ontario School of Medicine University, Sudbury, ON P3E 5J1, Canada

**Keywords:** glioma, glioblastoma, hypoxia, positron emission tomography, fluoromisonidazole, diacetyl-bis(N4-methylthiosemicarbazone)

## Abstract

**Background/Objectives:** While MRI is effective for glioma diagnosis, it has limitations in grading, treatment planning, and prognostication. Since hypoxia is associated with higher-grade gliomas and poorer outcomes, PET imaging with hypoxia-specific tracers has been shown to improve glioma assessment. This systematic review and meta-analysis aimed to evaluate the performance of hypoxia PET imaging in glioma diagnosis and prognostication. **Methods**: Systematic searches were conducted across PubMed, Web of Science, and Scopus through 31 January 2025. Only studies assessing the diagnostic or prognostic value of PET with ^18^F-labelled nitroimidazole (^18^F-FMISO, ^18^F-FAZA, ^18^F-FRP170, or ^18^F-FETNIM) or ^62^Cu-labelled ATSM hypoxia tracers in patients with gliomas were included. Hierarchical models were used to evaluate pooled performance on differentiating glioblastoma from lower-grade gliomas. **Results:** Thirty-eight articles (*n* = 1156 patients) were eligible for inclusion, and eleven articles (*n* = 296 patients) were suitable for meta-analytical calculations. The extent of hypoxia on PET imaging was generally correlated with isocitrate dehydrogenase (IDH) mutation status and histological angiogenesis. Hypoxia PET was effective at differentiating glioblastoma from lower-grade gliomas and at predicting overall and progression-free survival. In a pooled analysis, ^18^F-FMISO PET displayed high sensitivity (98%) and specificity (94%) for differentiating glioblastoma from lower-grade gliomas. **Conclusions:** Hypoxia PET has the potential to predict tumour biology, and it may be a reliable modality in combination with MRI to provide complementary information for glioma diagnosis, grading, treatment planning, and prognostication. It may be particularly useful for ruling out glioblastoma in patients with otherwise equivocal imaging; however, this would need to be validated prospectively with standardized image acquisition and interpretation criteria.

## Simple Summary

Gliomas are the most common class of malignant primary brain tumours, comprising a range of diagnoses with heterogeneous cellular origins, molecular hallmarks, prognoses, and treatments. Therefore, effectively diagnosing, classifying, and prognosticating glioma patients is critical for providing effective care. MRI is the gold standard imaging modality for diagnosing glioma, but it has limitations for grading, treatment planning, and predicting survival. This systematic review examines the evidence for the utility of hypoxia PET, an emerging technology that utilizes radioactive tracers which accumulate in tissues with low oxygen levels, in glioma patients. Hypoxia PET was effective at differentiating glioblastoma from lower-grade gliomas and at predicting overall and progression-free survival. In a pooled analysis of 201 patients across eight studies, the hypoxia PET tracer ^18^F-FMISO displayed high sensitivity (98%) and specificity (94%) for differentiating glioblastoma from lower-grade gliomas.

## 1. Introduction

Gliomas are a common class of primary brain neoplasms, accounting for approximately a quarter of all central nervous system (CNS) tumours [1]. They are highly heterogeneous in terms of molecular characteristics, treatment response, and overall prognosis, and are classified into four grades (I-IV) and various subtypes according to World Health Organization (WHO) criteria [2,3,4]. High-grade gliomas are associated with poor prognoses and treatment resistance. In particular, glioblastoma (WHO Grade IV) is almost uniformly fatal and has a median survival of approximately 14 months despite maximal treatment [5].

Magnetic resonance imaging (MRI) remains the preferred imaging modality for glioma diagnosis, providing critical anatomical information to help with identification, classification and for surgical planning [6]. However, conventional MRI has limitations in the context of gliomas, including a somewhat mediocre ability to differentiate tumour grades [7], a limited ability to assess or predict treatment response [8,9], and an unclear relationship with molecular features of the tumour and its microenvironment [10]. Although advanced MRI techniques, such as diffusion tensor imaging, MR spectroscopy, ultra-high-field MRI, and radiomics, can provide additional information about brain lesions, these techniques show heterogeneous results and lack widespread availability in clinical practice [6,11,12].

Positron emission tomography (PET) offers a complementary approach to interrogate the metabolic and molecular features of these tumours, including metabolism, proliferation, and grading [13]. Thus, it has the potential to complement MRI by providing functional information that can better inform treatment planning and prognostication. One key molecular feature of gliomas that can be assessed through PET imaging is hypoxia, which is a hallmark of aggressive glioma subtypes (particularly glioblastoma) [14,15]. Hypoxia-specific PET tracers, commonly based on ^18^F-labelled nitroimidazole or ^62^Cu-labelled ATSM, diffuse across cell membranes and, in low-oxygen environments, are reduced and retained intracellularly [16]. Measuring hypoxia through PET imaging could potentially aid in glioma diagnosis, grading, and prognostication. However, existing studies assessed a variety of tracers, study questions, imaging protocols, and patient cohorts, contributing to heterogeneity in findings and difficulty characterizing the benefits and limitations of this imaging modality.

This systematic review aims to provide updated, comprehensive data on the diagnostic and prognostic value of hypoxia PET in glioma patients. This includes the role of PET in evaluating hypoxia, as determined by immunohistochemical markers, as well as the clinical utility of hypoxia PET in the detection, grading, somatic mutation status prediction, treatment response monitoring, and survival prognostication of gliomas. This comprehensive literature review and meta-analysis highlights the strengths and limitations of hypoxia PET in glioma patients and identifies gaps in the current evidence base where future research is needed.

## 2. Methods

This study followed the recommendations of the Preferred Reporting Items for Systematic Review and Meta-Analysis of Diagnostic Test Accuracy studies (PRISMA-DTA) guidelines [17] (Supplemental File S1). The protocol has not been registered.

### 2.1. Search Strategy

A systematic search was conducted across three main medical literature libraries (PubMed/Medline, Web of Science, and Scopus) for all items published until 31 January 2025. The search was performed using database-specific Boolean search strategies, covering all key terms (text and MeSH terms) to include all studies of hypoxia using PET tracers for all gliomas. The core of the query string was as follows: [(“PET*” OR “positron”) AND (“hypoxia” OR “*FMISO*” OR “*fluoromisonidazole*” OR “*FAZA*” OR “*fluoroazomycin*” OR “*HX4*” OR “*flortanidazole*” OR “*ATSM*” OR “*methylthiosemicarbazone*” OR “*FETNIM*” OR”*EF1*” OR”*EF3*” OR “*EF5*” OR “*RP-170*” OR “*imidazole*”) AND (“*glioma*” OR “glioblastoma*” OR “GBM” OR “astrocytoma” OR “gliosarcoma” OR “glial cell”)].

### 2.2. Study Selection

Two independent reviewers (A.M.L. and S.A.M.) reviewed articles via EndNote. Discrepancies in article screening and data extraction were resolved through discussion. Only published original articles that met the following inclusion criteria were considered eligible:

1. Statistically evaluated the performance of hypoxia PET (using any ^18^F-labelled nitroimidazole or ^62^Cu-labelled ATSM tracers) at one or more of the following tasks:
  - Differentiating glioma from other CNS lesions, using histopathology as a reference standard
  - Predicting glioma grade, including differentiating high-grade gliomas from low-grade gliomas and differentiating glioblastoma from lower-grade gliomas, using histopathology as a reference standard
  - Predicting the status of specific gene mutations, including those for the isocitrate dehydrogenase genes (*IDH1* and *IDH2*), that are relevant for gliomas as outlined under the 2021 WHO guidelines
  - Predicting the expression of specific genetic or cellular markers, including CAIX, Ki67, HIF-1α, VEGF, and other markers related to hypoxia, metabolic changes, angiogenesis, or other relevant pathological processes
  - Predicting response to radiotherapy, neurosurgery, or systemic therapies such as chemotherapy or immunotherapy
  - Assessing response to radiotherapy, neurosurgery, or systemic therapies such as chemotherapy or immunotherapy
  - Prognosticating patient survival and/or time to progression.

We included studies that assessed PET alone, as well as PET/Computed Tomography (CT) and PET/MR studies.

When screening articles, the following exclusion criteria were used: scientific meeting abstracts, abstracts without full articles, reviews, preclinical studies on animal models, studies on synthetic images rather than acquired PET scans, studies which compare imaging modalities without a reference standard, studies which did not address any of the endpoints outlined in the inclusion criteria, and non-peer-reviewed/unpublished studies.

Studies which met inclusion criteria 1a and/or 1b above, as well as which provided adequate data to calculate the true positive (TP), true negative (TN), false positive (FP), and false negative (FN) results for these endpoints, were considered eligible for inclusion in a meta-analysis of diagnostic accuracy of hypoxia PET for glioblastoma.

Duplicate studies were removed from the dataset. Titles and abstracts were screened for relevance, followed by full-text review to determine eligibility for inclusion. The Quality Assessment of Diagnostic Accuracy Studies-2 (QUADAS-2) tool was utilized to assess the quality of diagnostic accuracy studies and exclude articles at high risk for bias [18]. Similarly, the Quality In Prognostic Studies (QUIPS) tool was used to assess the quality of prognostic studies and exclude articles at high risk of bias [19]. Studies that were deemed low-quality based on the above-mentioned tools and after consensus discussions were also excluded.

### 2.3. Data Extraction

Of the studies that were found to be eligible in full-text review, data extraction was performed (by A.M.L. and S.A.M. independently). Extracted data included: the first author’s name, year of publication, study outcome(s), reference standard, number of patients, imaging modality, PET tracer(s) used, diagnoses included, histopathological parameters (mutation status, gene expression, and/or immunohistochemical markers) collected, PET acquisition details, PET, PET/CT, or PET/MR device name, and PET interpretation criteria. For studies evaluating diagnostic performance, crude data (TP, FP, TN, FN) were extracted where available, as well as overall performance metrics (sensitivity, specificity, true positive rate, false positive rate, precision, recall, and/or accuracy).

### 2.4. Meta-Analytical Statistical Analysis

To analyze the pooled diagnostic performance of hypoxia PET for glioblastoma, the hierarchical method was employed to pool the random effect model’s measures of diagnostic metrics, including sensitivity, specificity, positive likelihood ratio (LR), and negative LR, from the derived two-by-two contingency tables. The pooled parameters were calculated to characterize a lesion as a glioblastoma. The bivariate model was employed to calculate the pooled sensitivity and specificity, along with their corresponding 95% confidence intervals (CI), to account for any variations within and across studies. The analyses were conducted using the STATA 16 (StataCorp, College Station, TX, USA) software module “Midas” [20].

## 3. Results

### 3.1. Study Characteristics

**Figure 1:**
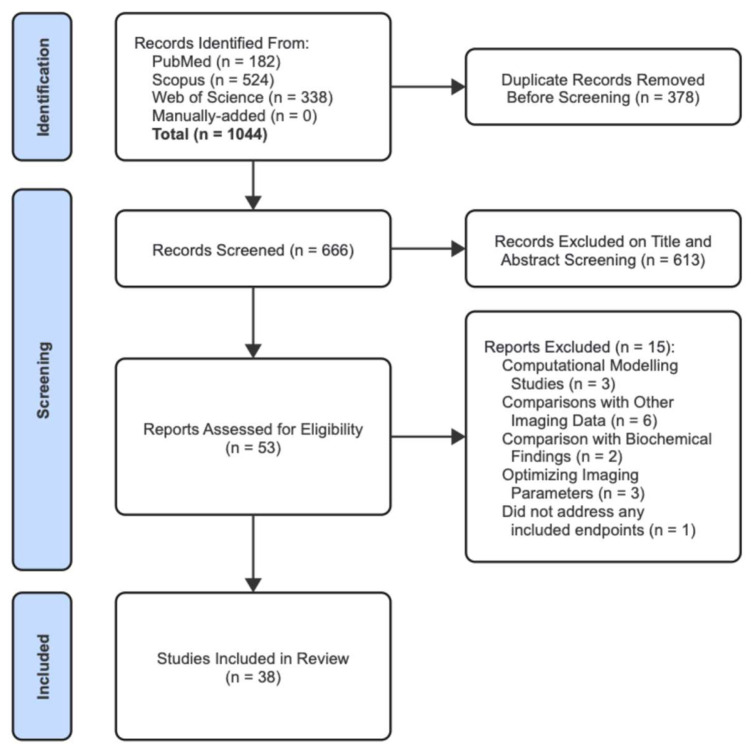
Study Selection Flowchart.

**Figure 2:**
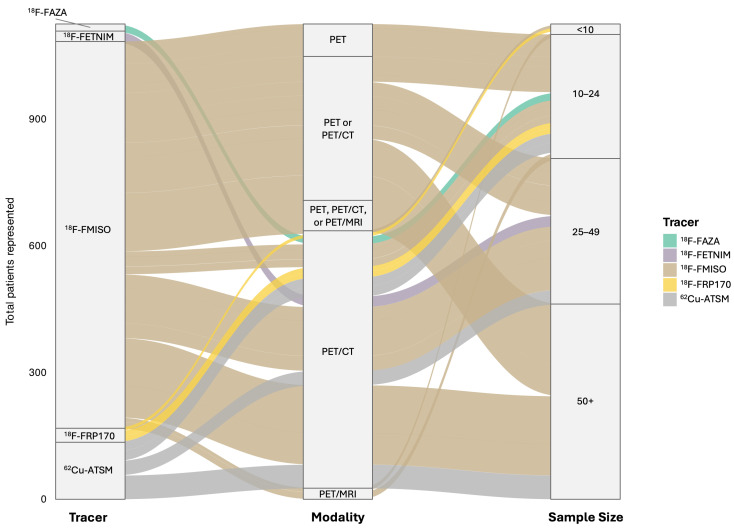
Alluvial diagram overview of included studies by PET tracer, imaging modality, and sample size. Colours indicate tracer, and band width indicates sample size. Figure created using ggalluvial version 0.12.6 [59].

Figure 1 illustrates the flow diagram for the selection process. The initial search resulted in 1044 articles. After removing duplicates, 666 articles underwent title and abstract screening, of which 613 articles were excluded. The remaining 53 underwent full-text review, and 38 studies (*n* = 1156 patients) were ultimately found to be eligible for the systematic review [21,22,23,24,25,26,27,28,29,30,31,32,33,34,35,36,37,38,39,40,41,42,43,44,45,46,47,48,49,50,51,52,53,54,55,56,57,58]. Considering the quality assessment, the main concerns for diagnostic accuracy studies were a risk of bias in patient selection (33% of applicable studies had a high risk of bias, and 56% had an unclear risk of bias) and in the applicability concerns of the index test (100% of studies had an unclear applicability), as studies differed greatly in their protocols, tracers, and scanners (Figure 2, Supplemental Table S1). For prognostic studies, there was often a risk of bias due to a lack of reporting surrounding study attrition (87% of studies did not report on attrition) and confounding factors (47% of studies did not report on confounding factors) (Supplemental Figure S1).

The included articles utilized a diversity of PET tracers, research questions, and imaging modalities (Table 1, Figure 2). Twenty-nine articles evaluated ^18^F-fluoromisonidazole (^18^F-FMISO) PET, one article evaluated ^18^F-fluoroazomycin arabinoside (^18^F-FAZA) PET, four articles evaluated ^62^Cu-diacetyl-bis (N4-methylthiosemicarbazone) (^62^Cu-ATSM) PET, one article evaluated ^18^F-fluoroerythronitroimidazole (^18^F-FETNIM) PET, and three articles evaluated 1-(2-fluoro-1-[hydroxymethyl]ethoxy)methyl-2-nitroimidazole (^18^F-FRP170) PET. Six studies provided data for differentiating glioma from other CNS entities, sixteen studies provided data on tumour grading, three studies provided data on gene mutation prediction, thirteen studies provided data on evaluating histopathological findings, seven studies provided data on assessing therapeutic responses, and sixteen studies provided data on patient survival prognostication. Five studies used PET imaging alone, twenty used PET/CT imaging alone, three used PET/MR imaging alone, nine used a combination of PET and PET/CT imaging, and one used a combination of PET/CT and PET/MR imaging. The performance of hypoxia PET at various clinical tasks is summarized in Table 2, Table 3 and Table 4.

### 3.2. Differentiating Glioma from Other Entities

#### 3.2.1. Systematic Review

Imaging plays an important role in differentiating gliomas from other intracranial masses. Six studies (*n* = 174 patients) assessed the performance of hypoxia PET for differentiating gliomas from other CNS lesions [26,28,31,38,45,55], including other primary CNS malignancies (e.g., germinoma, primary CNS lymphoma), benign non-glioma CNS tumours (e.g., meningioma), and metastases from primary tumours outside the CNS. Of these, five articles [26,28,38,45,55] studied ^18^F-FMISO PET, whereas only one studied ^62^Cu-ATSM PET [31]. The study details and performance characteristics are outlined in Table 2.

**Table 2:** Summary of studies assessing the performance of hypoxia PET for differentiating glioma from other diagnoses.

Paper	Tracer	No. of Patients	Included Diagnoses	Interpretation Criteria	Performance
Bruehlmeier 2004 [26]	^18^F-FMISO	11	Benign and Malignant Brain Tumours (Glioblastoma, Anaplastic Astrocytoma, Fibrillary Astrocytoma, Hemangioblastoma, Meningioma)	Dynamic imaging, with distribution volume > 1 at 150–170 min after tracer injection	Sensitivity = 66.6%, Specificity = 50% TP = 6, FN = 3, TN = 1, FP = 1
Cher 2006 [28]	^18^F-FMISO	16	Benign and Malignant Primary Brain Tumours (Grades I/II/III/IV Glioma) And Metastatic Brain Tumours	SUVmax; qualitative uptake	Sensitivity = 50%, Specificity = 0% TP = 7, FN = 7, FP = 2, TN = 0
Hino-Shishikura 2014 [31]	^62^Cu-ATSM	34	Gliomas and Primary CNS Lymphoma	SUVmax; Tumour-to-Background ratio	Significant differences in SUVmax and Tumour-to-Background ratio between PCNSL and both high- and low-grade gliomas (*p* < 0.0001 for LGG vs. PCNSL, *p* < 0.05 for HGG vs. PCNSL)
Kobayashi 2020 [38]	^18^F-FMISO	23	Benign and Malignant Brain Tumours (including gliomas, metastases, glial proliferation, and tumefactive demyelination)	Qualitative uptake	At 2 h: Sensitivity = 100%, Specificity = 0%. TP = 14, FP = 9, TN = 0, FN = 0 At 4 h: Sensitivity = 50%, Specificity = 100%. TP = 7, FP = 0, TN = 9, FN = 7
Shimizu 2020 [45]	^18^F-FMISO	15	Primary Brain Tumours (Glioma, Lymphoma, and Germinoma)	Lesion to cerebellum ratio >1.3	Sensitivity = 77%, Specificity = 50% TP = 10, TN = 1, FP = 1, FN = 3
Uchinomura 2022 [55]	^18^F-FMISO	75	Glioblastoma and Primary CNS Lymphoma	Qualitative uptake; Tumour-to-Normal ratio >1.75	Mean Tumour-to-Normal ratio is lower in PCNSL than in glioblastoma (*p* < 0.001)

^62^Cu-ATSM = ^62^Cu-diacetyl-bis (N4-methylthiosemicarbazone);^18^F-FMISO = ^18^F-fluoromisonidazole; CNS = Central Nervous System; FN = False Negative; FP = False Positive; HGG = High-Grade Glioma; LGG = Low-Grade Glioma; PCNSL = Primary Central Nervous System Lymphoma; SUVmax = maximum standardized uptake value; TN = True Negative; TP = True Positive.

Across the included studies, while specific subtypes of gliomas (e.g., glioblastoma) were typically positive for hypoxia tracers such as ^18^F-FMISO, several other subtypes, such as anaplastic astrocytoma (grade III), anaplastic oligodendroglioma (grade III), and fibrillary astrocytoma (grade II), often did not show sufficient tracer uptake [26,28,38]. The uptake of hypoxia tracers for non-glioblastoma gliomas differed between studies [26,28,38,45]. This may result from differences in tracer administration and PET timing, as Kobayashi et al. found that oligodendroglioma, anaplastic astrocytoma, diffuse astrocytoma and anaplastic oligodendroglioma patients were positive for ^18^F-FMISO uptake on visual assessment 2 h after tracer administration, but negative 4 h after administration [38]. Furthermore, a variety of non-glioma intracranial masses, including hemangioblastoma, metastatic adenocarcinoma, primary CNS lymphoma, and germinoma, have been shown to take up ^18^F-labelled nitroimidazole and/or ^62^Cu-labelled ATSM hypoxia PET tracers [26,28,45,55].

#### 3.2.2. Meta-Analysis

Next, given the repeated finding that glioblastoma was positive on hypoxia PET (Table 2), we sought to identify the diagnostic capabilities of hypoxia PET for glioblastoma diagnosis. We performed a pooled meta-analysis of all included studies, which evaluated both glioblastoma and other brain lesion etiologies (lower-grade gliomas, other primary brain tumours, or brain metastases) using hypoxia PET. 11 studies (*n* = 296 patients) provided sufficient data to be included in this meta-analysis [23,26,27,28,31,32,38,45,50,55,58], although this dataset was heavily biased towards studies evaluating solely gliomas rather than comparisons with non-glioma brain lesions. The pooled results suggest that hypoxia PET is reliable at excluding glioblastoma when negative (sensitivity = 97%, 95% CI: 89%, 99%), although specificity was lower (81%, 95% CI: 59%, 93%) (Supplemental Figure S2). The positive LR was 5.2 (95% CI: 2.1, 13.1), the negative LR was 0.03 (95% CI: 0.01, 0.15), and the diagnostic odds ratio was 168 (95% CI: 22, 1303). While there was some heterogeneity in the results, which may reflect a lack of standardization in imaging protocols and patient populations, this did not reach the threshold for statistical significance (Higgins I2 = 23% [95% CI: 0%, 100%]; Cochrane Q = 2.595, *p* = 0.137) (Supplemental Figure S2).

Altogether, these findings demonstrate that the value of hypoxia PET in differentiating all-grade gliomas from other CNS lesions is limited by heterogeneity in uptake between different glioma subtypes and in non-glioma brain lesions. However, hypoxia PET is highly sensitive for the identification of glioblastoma, although specificity is moderate.

### 3.3. Glioma Grading

#### 3.3.1. Systematic Review

Overall, sixteen studies (*n* = 456 patients) assessed the utility of hypoxia PET for differentiating between gliomas of different grades [23,26,27,28,31,32,33,35,37,38,41,44,45,50,51,58]. Of these, eleven assessed ^18^F-FMISO PET, three assessed ^62^Cu-ATSM PET, one assessed ^18^F-FETNIM PET, and one assessed ^18^F-FRP170 PET. The study characteristics, grading tasks, and performance are summarized in Table 3.

**Table 3:** Summary of studies assessing the performance of hypoxia PET for predicting glioma grade.

Paper	Tracer	No. of Patients	Grading Task	Interpretation Criteria	Performance
* Bekaert 2017 [23]	^18^F-FMISO	33	GBM vs. Non-GBM Glioma HGG vs. LGG	Hypoxic volume (volume with T/B Ratio >= 1.2) > 0 cm^3^	GBM vs. Non-GBM: Sensitivity = 96% and Specificity = 96% TP = 23, FP = 1, TN = 8, FN = 1 HGG vs. LGG: Sensitivity = 88%, Specificity = 83% TP = 23, FP = 1, FN = 3, TN = 5
*† Bruehlmeier 2004 [26]	^18^F-FMISO	9	GBM vs. Non-GBM Glioma HGG vs. LGG	Dynamic imaging, with distribution volume > 1 at 150–170 min after tracer injection	GBM vs. Non-GBM: Sensitivity = 100%, Specificity = 100% TP = 7, FP = 0, TN = 2, FN = 0 HGG vs. LGG: Sensitivity = 88%, Specificity = 100% TP = 7, FP = 0, TN = 1, FN = 1
* Chakhoyan 2017 [27]	^18^F-FMISO	13	GBM vs. Non-GBM Glioma	T/B ratio > 1.2	Sensitivity = 100%, Specificity = 100% TP = 10, TN = 3, FP = 0, FN = 0
*† Cher 2006 [28]	^18^F-FMISO	14	GBM vs. Non-GBM Glioma; HGG vs. LGG	SUVmax; qualitative uptake	GBM vs. Non-GBM: Sensitivity = 100%, Specificity = 100%, TP = 7, FP = 0, TN = 7, FN = 0 HGG vs. LGG: Sensitivity = 70%, specificity = 100%, TP = 7, FN = 3, TN = 4, FN = 0
*† Hino-Shishikura 2014 [31]	^62^Cu-ATSM	34	GBM vs. Non-GBM Glioma	SUVmax; T/B ratio	GBM had significantly higher SUVmax and T/B ratio than LGG (*p* < 0.0001 for both)
* Hirata 2012 [32]	^18^F-FMISO	23	GBM vs. Non-GBM Glioma	Qualitative uptake	Sensitivity 100%, Specificity 100% TP = 14, TN = 9, FP = 0, FN = 0
Hu 2020 [33]	^18^F-FETNIM	25	GBM vs. Non-GBM Glioma	SUVmax	Significant difference in SUVmax of GBM vs. grade III and grade II gliomas (*p* = 0.007 and *p* < 0.001 respectively); Pearson correlation (r = 0.881, *p* < 0.001) of grade with SUVmax
Kanoto 2018 [35]	^18^F-FMISO	41	HGG vs. LGG	T/Nmax > 1.25; T/Nmean > 1.23	T/Nmax: sensitivity = 90%, specificity = 90.9% T/Nmean: sensitivity = 93.3%, specificity = 90.9%
Kawai 2014 [37]	^18^F-FMISO	32	GBM vs. Grade III Glioma	Either HV (volume with T/B > 1.2) or T/Bmax	Significant difference in mean HV and T/Bmax between GBM and Grade III Glioma (*p* < 0.01 for both)
*† Kobayashi 2020 [38]	^18^F-FMISO	23	GBM vs. Non-GBM Glioma	Visual Assessment; T/Bmax (>1.51)	Visual Assessment: Sensitivity = 100%, Specificity = 100%, TP = 7, TN = 16; FP = 0, FN = 0 T/Bmax: Sensitivity = 100%, Specificity = 100%, TP = 7, TN = 16; FP = 0, FN = 0
Miyake 2021 [41]	^18^F-FMISO	113	GBM vs. non-GBM Glioma	T/Nmax	Significant difference (*p* < 0.001) between GBM and non-GBM
Shibahara 2010 [44]	^18^F-FRP170	8	GBM vs. Non-GBM	SUVmax	Higher FRP170 SUVmax for all GBM patients than for all non-GBM patients
*† Shimizu 2020 [45]	^18^F-FMISO	13	GBM vs. Non-GBM HGG vs. LGG	Tumour-to-Cerebellum ratio > 1.3	GBM vs. Non-GBM: Sensitivity = 100%, specificity = 40%, TP = 7, TN = 2, FP = 3, FN = 0 HGG vs. LGG: Sensitivity = 100%, specificity = 100%, TP = 10, TN = 2, FP = 0, FN = 0
* Tateishi 2013 [50]	^62^Cu-ATSM	22	GBM vs. Grade III Glioma	Directly Reported SUVmax, SUVmean +/− SD, and T/B Ratio	Significant Difference in Mean SUVmax and SUVmean between GBM and Grade III Glioma (*p* = 0.014 and *p* =0.010, respectively) Using a T/Bmax cutoff of 1.9, 90.9% sensitivity and 90.9% specificity for differentiating GBM from lower grade glioma, TP = 10; FP = 1; TN = 10; FN = 1
Tateishi 2014 [51]	^62^Cu-ATSM	23	GBM vs. Non-GBM Glioma	T/B ratio > 1.9	90% sensitivity, 76.9% specificity
* Yamamoto 2012 [58]	^18^F-FMISO	30	GBM vs. Non-GBM Glioma; HGG vs. LGG	GBM vs. NonGBM: T/Bmax > 1.97 HGG vs. LGG: T/Bmax > 1.2	GBM vs. non-GBM: TP = 15, FP = 2, TN = 12, FN = 1 GBM vs. LGG: sensitivity = 100%, specificity = 100%

^62^Cu-ATSM = ^62^Cu-diacetyl-bis (N4-methylthiosemicarbazone); ^18^F-FETNIM = ^18^F-fluoroerythronitroimidazole; ^18^F-FMISO = ^18^F-fluoromisonidazole; ^18^F-FRP170 = 1-(2-fluoro-1-[hydroxymethyl]ethoxy)methyl-2-nitroimidazole; FN = False Negative; FP = False Positive; GBM = glioblastoma; HGG = High-Grade Glioma; HV = Hypoxic Volume; LGG = Low-Grade Glioma; SUVmax = maximum standardized uptake value; SUVmean = mean standardized uptake value; T/B = Tumour-to-Background Ratio; T/Bmax = Tumour-to-Background SUVmax; T/N = Tumour-to-Normal Ratio; T/Nmax = Tumour-to-Normal SUVmax; T/Nmean = Tumour-to-Normal SUVmean; TN = True Negative; TP = True Positive. * = studies included in meta-analysis. † = studies for which data on non-glioma patients (e.g., lymphoma) were removed for the meta-analysis.

Nine studies included sufficient data to assess the accuracy of hypoxia PET for differentiating high-grade (grade III/IV) gliomas from low-grade (grade I/II) gliomas [23,26,28,31,33,35,45,50,58]. The accuracy of hypoxia PET at differentiating high- and low-grade gliomas differed between studies (Table 3). For instance, Bekaert et al. found that a Tumour/Background ratio >1.2 was 88% sensitive and 83% specific for differentiating high-grade gliomas, whereas Yamamoto and colleagues found this same threshold to be 100% sensitive and 100% specific for this task [23,58]. Differences between studies may be related to evaluated thresholds for tracer uptake and/or the timing between the injection of ^18^F-FMISO, as Kobayashi et al. demonstrated that grade III gliomas were positive for ^18^F-FMISO after 2 h, but negative after 4 h [38]. This suggests that the accuracy of hypoxia PET for differentiating high- from low-grade gliomas may depend on the time delay between tracer injection and imaging.

In contrast, hypoxia PET was highly accurate at differentiating glioblastoma from other gliomas (Table 3) across all fifteen included studies that included sufficient data to assess this [23,26,27,28,31,32,33,35,37,38,41,45,50,51,58]. Eleven of these studies used ^18^F-FMISO, three used ^62^Cu-ATSM, and one used ^18^F-FETNIM. Five studies reported a 100% sensitivity and 100% specificity in differentiating glioblastoma from lower-grade gliomas [26,27,28,32,38]. However, other studies report lower estimates of sensitivity and specificity for differentiating glioblastoma from low-grade glioma [23,51,58]. Studies which focused on quantitative differences in hypoxia PET parameters between glioblastoma and lower-grade gliomas, rather than diagnostic performance parameters such as sensitivity and specificity, similarly found significantly higher hypoxia tracer uptake in glioblastomas [31,37,41,50,51].

#### 3.3.2. Meta-Analysis

**Figure 3:**
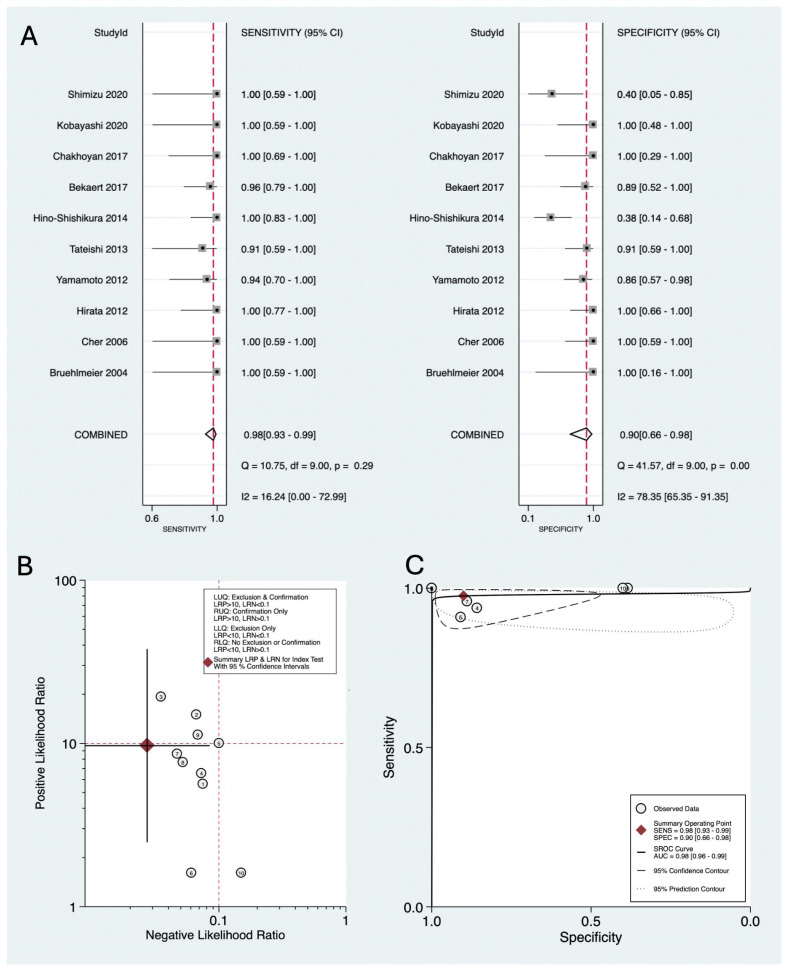
Overview of meta-analysis results for the diagnostic value of hypoxia PET (all tracers) for distinguishing glioblastoma from lower-grade gliomas. (**A**) Forest plots for the pooled sensitivity and specificity calculation for differentiating glioblastoma from lower-grade gliomas. Horizontal lines represent 95% confidence intervals of the individual studies. The dashed red line is the line of summary points across the studies (**B**) Likelihood ratio scattergram for glioblastoma assessment (**C**) Hierarchical summary receiver operating characteristic (SROC) curve for overall response assessment. The “observed data” points show accuracy for each study, and the “summary operating point” represents the pooled accuracy [23,26,27,28,31,32,38,45,50,58]. AUC = area under the receiver operating characteristic curve, LLQ = left lower quadrant, LRN = negative likelihood ratio, LRP = positive likelihood ratio, LUQ = left upper quadrant, RLQ = right lower quadrant, RUQ = right upper quadrant, SENS = sensitivity, SPEC = specificity, SROC = summary receiver operating characteristic curve.

**Figure 4:**
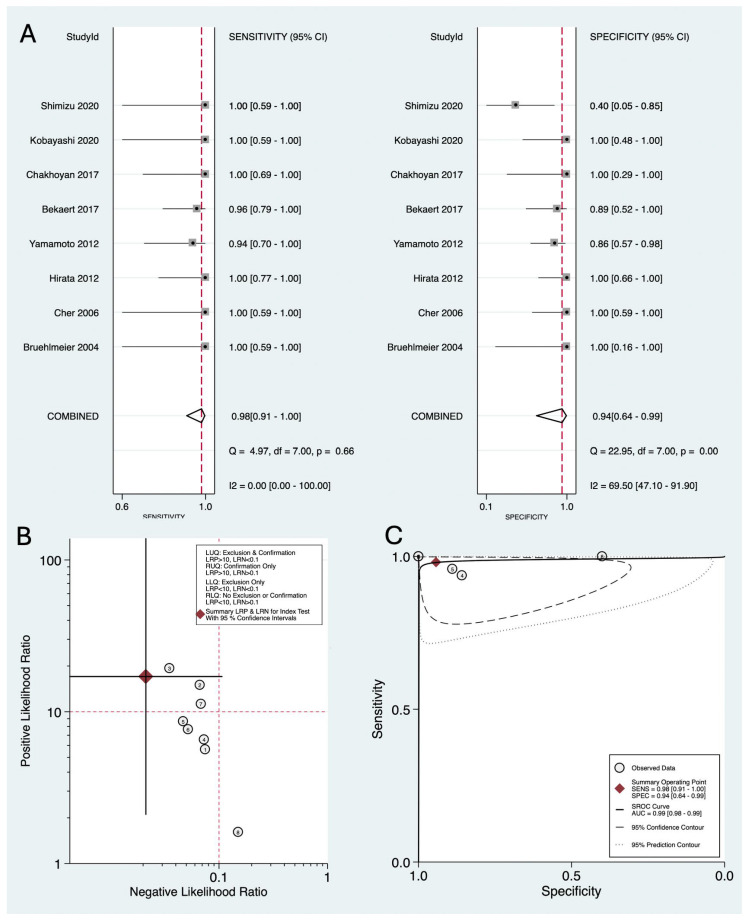
Overview of meta-analysis results for the diagnostic value of ^18^F-FMISO PET for distinguishing glioblastoma from lower-grade gliomas. (**A**) Forest plots for the pooled sensitivity and specificity calculation for differentiating glioblastoma. Horizontal lines represent 95% confidence intervals of the individual studies. The dashed red line is the line of summary points across the studies (**B**) Likelihood ratio scattergram for glioblastoma assessment (**C**) Hierarchical summary receiver operating characteristic (SROC) curve for overall response assessment. The “observed data” points show accuracy for each study, and the “summary operating point” represents the pooled accuracy [23,26,27,28,32,38,45,58]. AUC = area under the receiver operating characteristic curve, LLQ = left lower quadrant, LRN = negative likelihood ratio, LRP = positive likelihood ratio, LUQ = left upper quadrant, RLQ = right lower quadrant, RUQ = right upper quadrant, SENS = sensitivity, SPEC = specificity, SROC = summary receiver operating characteristic curve.

We performed a pooled meta-analysis of all studies with sufficient data to evaluate the sensitivity and specificity of hypoxia PET in distinguishing glioblastoma from lower-grade gliomas. Ten studies (*n* = 256 patients) had sufficient data for inclusion in this meta-analysis [23,26,27,28,31,32,38,45,50,58]. Overall, the pooled sensitivity was 98% (95% CI: 93%, 99%) and the pooled specificity was 90% (95% CI: 66%, 98%) for differentiating glioblastoma from lower tumour grades (Figure 3). The positive LR was 9.7 (95% CI: 2.5, 37.6), the negative LR was 0.03 (95% CI: 0.01, 0.08), and the diagnostic odds ratio was 356 (95% CI: 58, 2197). However, there was significant heterogeneity in the results (Higgins I^2^ = 59% [95% CI: 8%, 100%]; Cochran Q = 4.924, *p* = 0.043). Given the differences in kinetics and uptake mechanisms between different hypoxia PET tracers [60], we performed an additional meta-analysis that limited the data studies to only ^18^F-FMISO (Figure 4). Across eight included studies (*n* = 201 patients), the pooled meta-analysis demonstrated a sensitivity of 98% (95% CI: 91%, 100%) and specificity of 94% (95% CI: 64%, 99%) for differentiating glioblastoma from lower-grade gliomas. The positive LR was 17.1 (95% CI: 2.1, 138.1), the negative LR was 0.02 (95% CI: 0.00, 0.11), and the diagnostic odds ratio was 804 (95% CI: 39, 16529). Furthermore, there was no significant heterogeneity in these ^18^F-FMISO-only results (Higgins I^2^ = 0, Cochrane Q = 0.831, *p* = 0.330). Additionally, we investigated report bias in this rather homogeneous dataset, and the analysis suggested symmetry in the data and a low likelihood of publication bias (*p* = 0.44; Supplemental Figure S3).

The significant heterogeneity observed in the all-tracer pooled analysis (I^2^ = 59%) was likely driven by the inclusion of non−^18^F-FMISO tracers, as evidenced by the complete resolution of heterogeneity upon restriction to ^18^F-FMISO studies (I^2^ = 0%). However, given that only two studies utilized non-FMISO tracers in this meta-analysis [31,50], it was not feasible to perform a statistically meaningful subgroup analysis of these agents independently.

In summary, these results suggest that hypoxia PET in general, and ^18^F-FMISO in particular, performs well at differentiating glioblastoma from lower-grade gliomas.

### 3.4. Gene Mutation Prediction

Mutations in the isocitrate dehydrogenase (IDH) genes and 1p/19q codeletion are important molecular features in the 2021 WHO categorization of brain tumours [2,3]. Three included studies assessed the correlation of hypoxia PET findings with these molecular features [41,47,56]. All three studies examined ^18^F-FMISO PET. Wang et al. found that relative SUVmax and SUVmean of ^18^F-FMISO PET were significantly higher in IDH-wildtype high-grade gliomas than IDH-mutant gliomas (*p* < 0.05) [56]. Similarly, Suzuki et al. found that the median Tumour-to-Blood ratio from ^18^F-FMISO PET had a sensitivity of 81% and a specificity of 65% for predicting IDH mutation status [47]. When performing subgroup analyses, the ability of FMISO uptake to differentiate between IDH-mutant and IDH-wildtype tumours was not present in anaplastic astrocytomas—suggesting that the ability of hypoxia to predict IDH mutation status may differ by specific glioma type. Miyake and colleagues found that ^18^F-FMISO PET was effective at differentiating glioblastoma from the other glioma subtypes, and that a combination of FMISO PET findings with MRI features was able to differentiate between IDH-wildtype and IDH-mutant astrocytomas [41].

In summary, the limited data from these three studies suggest that ^18^F-FMISO PET, especially when combined with features on MRI, may be an effective modality for predicting the IDH mutation status of gliomas.

### 3.5. Gene Expression Evaluation

Tissue hypoxia is typically defined based on the differential expression of genes involved in the response to hypoxia. Thirteen studies (*n* = 282 patients) compared hypoxia tracer uptake with gene expression data, evaluated with either immunohistochemistry or PCR-based gene expression quantification [23,24,25,28,33,36,37,40,44,46,48,50,53]. One paper assessed ^18^F-FAZA, one paper assessed ^62^Cu-ATSM, one paper assessed ^18^F-FETNIM, three papers assessed ^18^F-FRP170, and the remaining seven papers assessed ^18^F-FMISO PET (Supplemental Table S2). The most commonly assessed markers were Hypoxia-Inducible Factor 1-Alpha (HIF-1α, in 9 studies), a key marker of the cellular response to hypoxia, Ki-67 (in 5 studies), a nuclear protein used to quantify cellular proliferation, and vascular endothelial growth factor (VEGF, in 5 studies), a marker for angiogenesis. However, a variety of other genetic markers were examined across studies, including CA-IX [23,40,48], Glucose Transporter 3 [28], VEGF-R [28], Ang2 [23], MMP-9 [33], PD-L1 [48], and p53 [46], as well as other pathological markers including tumour vascularity [28,40] and pathological necrosis [53].

Across the included studies, there was considerable variability in the correlation of tracer uptake with immunohistochemical staining for HIF-1α, a key marker of the cellular response to hypoxia. While some studies reported a significant correlation [23,25,33,50], others found no such correlation [28,37,46]. Of the four papers which reported a statistically significant connection between HIF-1α and tracer uptake—either as a correlation or as a predictor for the presence or absence of HIF-1α staining—only one paper used ^18^F-FMISO PET, whereas the papers that reported no significant correlations all used ^18^F-FMISO PET. Similarly, papers differed in whether hypoxia tracer uptake was related to immunostaining for Ki-67, a marker of cellular proliferation [25,28,33,36,46]. There was also a high degree of heterogeneity in methods used to score the positivity of histological slides for HIF-1α and Ki-67, limiting the generalizability of these findings [23,24,25,28,33,36,37,44,46,50].

CA-IX, a negative prognostic marker and hypoxia response protein in glioblastoma, was significantly correlated with hypoxia tracer uptake in all three papers that assessed it [23,40,48]. Several other markers, including Ang2 [23], MMP-9 [33], and PD-L1 [48], as well as histological features such as micro-necrosis [53], correlated with hypoxia tracer uptake, although each was only assessed in one paper.

While some studies reported conflicting findings [46], the angiogenesis markers VEGF and VEGF-R were often correlated with hypoxia tracer uptake [23,28,33,37]. This suggests that hypoxia tracer uptake may broadly reflect an angiogenic microenvironment, which may be relevant in deciding on and monitoring antiangiogenic therapies such as bevacizumab (see Assessing Responses to Therapy) below.

In summary, hypoxia tracer uptake is generally correlated with markers of angiogenesis such as VEGF and cellular responses to hypoxia, but the extent to which it correlates with HIF-1α and Ki-67 significantly varies between studies.

### 3.6. Assessing Responses to Therapy

Hypoxia PET may be helpful in monitoring how tumours change and respond over the course of treatment. Seven included studies assessed the value of hypoxia PET in monitoring treatment response and disease progression across a variety of treatment modalities [21,22,30,39,48,49,57]. All seven studies used ^18^F-FMISO to assess hypoxia.

Checkpoint inhibitors such as pembrolizumab are increasingly utilized treatments for glioblastoma, but it can be challenging to distinguish treatment failure and disease progression from ‘pseudoprogression’, or the appearance of progression on imaging despite pathologic treatment success. Barajas et al. demonstrated that ^18^F-FMISO PET/MRI was able to distinguish between these two scenarios; however, only in a small cohort of six glioblastoma patients treated with pembrolizumab, as the mean hypoxic fraction of pseudoprogression was significantly smaller than that of tumour recurrence and treatment failure [22].

Another systemic therapy used to treat glioblastoma, for which hypoxia PET may be helpful in delineating responses to therapy, is the anti-VEGF agent bevacizumab. Several included studies demonstrate that ^18^F-FMISO PET is able to visualize tumour responses to bevacizumab [21,48,57]. For example, Yamaguchi and colleagues found that patients who responded to bevacizumab (as measured by ^18^F-FMISO) had significantly longer survival times than those who did not (median 12.4 vs. 5.7 months, *p* < 0.001) [57].

Beyond systemic therapy, other studies have assessed the efficacy of hypoxia PET to monitor the response of patients to treatment with surgery and/or radiotherapy. Two included studies assessed the value of serial imaging with hypoxia PET in monitoring lesions before and after local treatment and found that hypoxic volume often decreases after treatment [30,49]. Furthermore, early treatment response as detected by hypoxia PET may also predict long-term responses to therapy; Leimgruber et al. found that increases in hypoxic volume after radiotherapy were associated with significantly shorter time to disease progression in glioblastoma patients [39].

Overall, hypoxia PET may be a promising method to evaluate responses to systemic and local cancer treatments, but the existing data is from a limited number of studies with small cohorts.

### 3.7. Patient Survival Prognostication

Imaging is a critical tool in supporting clinicians when estimating the prognosis of patients with brain tumours. Fifteen studies (*n* = 473 patients) assessed the prognostic value of hypoxia PET [23,28,29,30,33,34,37,39,42,43,46,49,52,54,57], with all fifteen studies examining overall survival (OS) and nine studies [23,28,30,34,39,42,46,52,54] examining progression-free survival (PFS) or time to progression (TTP). Thirteen studies assessed ^18^F-FMISO, one study assessed ^18^F-FETNIM, and one study assessed ^62^Cu-ATSM. These studies are summarized in Table 4.

**Table 4:** Summary of studies assessing the performance of hypoxia PET for predicting time to progression, progression-free survival, and overall survival.

Paper	Tracer	No. of Patients	Included Diagnoses	Relationship of Hypoxia with Prognosis
Bekaert 2017 [23]	^18^F-FMISO	33	Glioma	Significantly shorter OS (*p* = 0.001) and PFS (*p* = 0.003) in patients with HV > 0
Cher 2006 [28]	^18^F-FMISO	14	Glioma	1-year OS was 43% in patients with positive uptake vs. 100% in patients with negative uptake; 1-year PFS was 0% in patients with positive uptake vs. 86% in patients with negative uptake.
Chvetsov 2024 [29]	^18^F-FMISO	22	Glioblastoma	HV was significantly correlated with OS (R^2^ = 0.39, *p* < 0.01); Equivalent uniform aerobic dose (EUAD) was significantly correlated with OS, both with (R^2^ = 0.54, *p* < 0.001) and without HV correction (R^2^ = 0.60, *p* < 0.001)
Gerstner 2016 [30]	^18^F-FMISO	42	Glioblastoma	Higher ^18^F-FMISO SUVpeak (*p* = 0.048) was significantly associated with shorter overall survival time; ^18^F-FMISO SUVpeak and HV were not significantly correlated with progression-free survival
Hu 2020 [33]	^18^F-FETNIM	25	Glioma	Patients with SUVmax > 1.68 had a significantly lower 3-year OS than those with SUVmax <= 1.68 (24.4% vs. 70.3%, *p* = 0.003)
Huang 2021 [34]	^18^F-FMISO	33	Glioblastoma	Progression-free survival decreased with hypoxic volume (HR = 1.67, *p* = 0.009); Overall survival decreased with hypoxic volume (HR = 1.71, *p* = 0.01)
Kawai 2014 [37]	^18^F-FMISO	32	High-Grade Glioma	Large (>= 20 cm^3^) HV associated with a shorter lifespan than a small (<20 cm^3^) HV (*p* < 0.01); T/Bmax >= 2.5 associated with a shorter lifespan than T/Bmax < 2.5 (*p* < 0.05)
Leimgruber 2019 [39]	^18^F-FMISO	10	Glioblastoma	Shorter TTP for patients with increased HV after radiotherapy (mean = 2.8 months) than those without (mean = 5.9 months); Hypoxic Volume significantly correlated with TTP (*p* < 0.01)
Muzi 2015 [42]	^18^F-FMISO	38	Glioma	T/Bmax > median was a significant predictor of survival and time to progression (*p* < 0.001) in pretreatment glioma patients
Muzi 2020 [43]	^18^F-FMISO	72	High-Grade Glioma	SUVpeak, SUVmean, and HV were all significantly correlated with overall survival (*p* < 0.001)
Spence 2008 [46]	^18^F-FMISO	22	Glioblastoma	Shorter time to progression and overall survival in patients with HV > 0 cm^3^ (*p* < 0.001); Shorter time to progression and overall survival in patients with T/Bmax > median (*p* < 0.001); Significant correlation of HV or T/Bmax with shorter TTP or OS (*p* < 0.002)
Swanson 2009 [49]	^18^F-FMISO	24	Glioblastoma	HV and HV surface area (HVsa) were significant predictors of survival (*p* < 0.05) in pre-operative patients, but not in post-operative and post-radiotherapy patients
Toriihara 2018 [52]	^62^Cu-ATSM	56	Glioma	SUVmax >= 1.5 was associated with shorter overall survival than SUVmax > 1.5 (*p* < 0.001); SUVmax >= 1.3 was associated with shorter progression-free survival than SUVmax < 1.3 (*p* < 0.001); SUVmax was inversely correlated with both overall survival (*p* < 0.05) and progression-free survival (*p* < 0.05)
Toyonaga 2016 [54]	^18^F-FMISO	32	Glioblastoma	Higher HV associated with significantly shorter overall survival (*p* = 0.04, HR = 4.65), but not progression-free survival (*p* > 0.05)
Yamaguchi 2016 [57]	^18^F-FMISO	18	Glioma	Bevacizumab response with both FMISO PET and MRI was associated with a longer OS than all other patients (median 12.4 vs. 5.7 months; *p* < 0.001)

^62^Cu-ATSM = ^62^Cu-diacetyl-bis (N4-methylthiosemicarbazone); ^18^F-FETNIM = ^18^F-fluoroerythronitroimidazole; ^18^F-FMISO = ^18^F-fluoromisonidazole; EUAD = equivalent uniform aerobic dose; HV = Hypoxic Volume; HVsa = Hypoxic Volume surface area; OS = Overall Survival; PFS = Progression-Free Survival; SUVmax = maximum standardized uptake value; SUVmean = mean standardized uptake value; T/Bmax = Tumour-to-Background SUVmax; TTP = Time to Progression.

Hypoxic volume, typically defined as the total volume in cm^3^ with a tumour-to-background ratio > 1.2, was repeatedly correlated with significantly shorter OS, including in cohorts of all-grade glioma, high-grade glioma, and glioblastoma patients [23,29,34,37,43,46,49,54]. However, the correlation of hypoxic volume with PFS was more variable; while most studies report a strong relationship [23,34,39,46], others did not find any significant relationship [30,54]. Other variables from hypoxia PET, such as SUVpeak [30,43], SUVmax [33,52], Tumour/Background SUVmax [37,42,46], have also been shown to correlate with shorter overall survival. Similarly, SUVmax [52] and Tumour/Background SUVmax [42,46], but not SUVpeak [30], have been found to correlate with progression-free survival.

Five studies specifically assessed the prognostic value of hypoxia PET after treatment [28,33,34,39,43]. One of these studies used ^18^F-FETNIM PET, whereas the remaining four studies assessed ^18^F-FMISO PET. Three of the included studies used hypoxia PET to predict survival in patients after surgery [28,33,43], one assessed the antiangiogenic agent bevacizumab [34], and one assessed the response to radiotherapy [39]. Across all of these studies, hypoxia tracer uptake was associated with shorter overall and shorter progression-free survival (Table 4).

Altogether, these results suggest that hypoxia tracer uptake (most commonly defined using hypoxic volume) is likely a negative prognostic factor in glioma and glioblastoma.

## 4. Discussion

In this study, we summarized and analyzed the current evidence for the diagnostic and prognostic value of hypoxia PET, with the meta-analysis focusing on tracers based on ^18^F-labelled nitroimidazole or ^62^Cu-labelled ATSM, in patients with different classes of glioma. In a pooled meta-analysis, hypoxia PET tracers showed high sensitivity and specificity for differentiating glioblastoma from lower-grade gliomas (see Results for pooled estimates). Furthermore, hypoxia PET tracers were partly correlated with histopathological and genetic markers used to characterize angiogenesis and hypoxia, although there was some heterogeneity in correlations with HIF-1α and Ki-67. While the evidence for the prognostic value of hypoxia PET for gliomas remains heterogeneous, higher levels of hypoxia tracer uptake were generally associated with shorter overall and progression-free survival, including in cohorts of glioblastoma only and cohorts with gliomas of multiple grades. Hypoxia PET may also be able to assess responses to therapies, including radiotherapy, bevacizumab, and immune checkpoint inhibitors, although the evidence for this is preliminary and heterogeneous.

Several imaging modalities, including MRI, ^18^F-FDG PET, and ^11C^-MET PET, have been shown in previous clinical studies and meta-analyses to be valuable for differentiating gliomas from other intracranial masses [61,62,63]. One particular challenge for conventional neuroimaging is differentiating gliomas from brain metastases [64], but advanced MRI techniques such as perfusion MRI and Neurite Orientation Dispersion and Density Imaging (NODDI) have been shown in systematic reviews to accurately differentiate between these entities [63,65]. Likewise, GBM can have a similar imaging appearance to primary CNS lymphoma on conventional MRI, but ^18^F-FDG PET/CT has been shown to accurately distinguish between these two entities in a recent systematic review [62]. In this context, the findings of our meta-analysis demonstrate that hypoxia PET has limited utility for differentiating gliomas from other intracranial masses, in part because the presence of hypoxia in gliomas was highly dependent on grade. In contrast, our meta-analytical results suggest that when specifically differentiating GBM from other intracranial masses, hypoxia PET offers high sensitivity (98%). However, specificity varies (with pooled estimates in glioma cohorts of 90% for all tracers and 94% for ^18^F-FMISO), as highly hypoxic entities such as PCNSL remain difficult to distinguish from GBM. Therefore, hypoxia PET may serve as a complementary imaging modality alongside MRI and FDG-PET, particularly to help rule out glioblastoma in cases where standard imaging is equivocal.

In terms of distinguishing glioma grades, previous studies and meta-analyses have reported moderate accuracy for advanced MRI methods as well as PET imaging with different amino acid tracers, including ^18^F-FDOPA and ^11^C-MET [6,66,67,68,69]. While advanced MRI techniques such as perfusion MRI and imaging-based machine learning techniques have demonstrated reasonable accuracy at differentiating glioblastoma from lower-grade gliomas, their diagnostic accuracy is typically lower than what was found for hypoxia PET in our meta-analysis [70,71,72]. Furthermore, existing MRI and PET methods (particularly amino acid PET and MR spectroscopy) perform well when distinguishing all high-grade (grade III/ IV) gliomas from low-grade (I/II) tumours [66,67,69], whereas hypoxia PET appears to perform best when differentiating glioblastoma in particular. Hypoxia PET may provide more specific information related to the distinct hypoxic microenvironment characteristic of glioblastoma. Thus, there might be a complementary role for hypoxia PET alongside other established imaging biomarkers. Given that clinical management and prognosis differ substantially between grade III gliomas and glioblastoma [73], the ability of hypoxia PET to refine this distinction could have meaningful diagnostic and therapeutic implications.

With respect to prognostic value, we found that an increase in hypoxia as assessed on PET was typically associated with worse OS and PFS, although there was some heterogeneity in the included studies in terms of prognostic parameters, imaging techniques, and results. Other emerging PET tracers have also been suggested as prognostic biomarkers in glioma patients. For instance, ^18^F-FET PET has repeatedly been shown to predict worse OS and PFS in patients with glioma [74,75,76]. However, many of these studies have focused on dynamic PET imaging, and comparative studies have demonstrated that dynamic amino acid PET is able to predict survival when static amino acid PET is not [74]. In contrast, there is limited data available on the prognostic value of dynamic hypoxia PET, indicating a need for further research in this area.

Current society guidelines, including the PET-based response assessment criteria for diffuse gliomas (PET RANO) [10] and EANM/EANO/RANO/SNMMI guidelines for amino acid PET [77], recognize the utility of novel tracers but lack guidance or suggestions regarding specific clinical uses of hypoxia PET. Likewise, a standardized set of protocols, tracers, or interpretation criteria for hypoxia PET in glioma does not yet exist, and relatively few large-scale, prospective clinical trials have been carried out on the diagnostic and prognostic value of this technique [30,78]. The high sensitivity seen in our pooled analysis suggests that hypoxia PET may be particularly useful for ruling out glioblastoma in patients where other modalities and tracers are otherwise equivocal. Given the heterogeneity in tracers and protocols in our study, as well as small sample sizes with unclear patient selection (Supplemental Table S1), further research is necessary. Ideally, further multi-centre, prospective, comparative studies can evaluate a range of tracers, including those which have only been recently made available, and include comparisons with other emerging modalities (e.g., amino acid PET, MR spectroscopy) to clarify the clinical utility of hypoxia PET for evaluating gliomas.

This systematic review had several limitations. The eligible studies for inclusion, including the subset used for the meta-analysis, had highly heterogeneous base data in terms of tracers, scanners, modalities (PET vs. PET-CT vs. PET-MRI), and protocols, limiting the generalizability of our conclusions about accuracy. This is further demonstrated by Kobayashi et al., who found that the diagnostic accuracy of ^18^F-FMISO in grading gliomas is dependent on the time from tracer injection to imaging [38]. Furthermore, a significant proportion of the included studies had a high or unclear risk of bias and/or concerns with applicability (Supplemental Figure S1). Additionally, due to insufficient data availability, we could not perform subgroup calculations to pool performance based on tracers, timepoint, or modality (PET alone vs. PET/CT vs. PET/MRI) for our meta-analysis. Given that it is unclear whether there are tracer-specific differences in the relationship of imaging findings to tumour biology, this limits the generalizability of our results. Low data availability and heterogeneity also obviated the possibility of performing additional meta-analyses for other clinical tasks such as survival prognostication.

## 5. Conclusions

In summary, hypoxia PET was found to be highly sensitive and specific for characterizing lesions as glioblastoma, with ^18^F-FMISO PET displaying accuracy that rivals advanced MRI techniques [66,70,71,72]. However, as the included studies were largely retrospective and had inconsistent criteria for image interpretation, prospective evaluation with standardized interpretation criteria can help validate this technique and facilitate its implementation into clinical neuro-oncology practice. Hypoxia tracers may also be useful for providing information about the underlying biology of tumours, and limited data suggest that they may play a role in treatment planning, monitoring, and prognosis.

## Figures and Tables

**Table 1:** Summary of Key Study Characteristics.

First Author and Year	Included Diagnoses and No. of Patients	Tracer and Imaging Modality	Relevant Outcome(s) Evaluated or With Sufficient Data to Evaluate	Diagnostic Reference Standard
Barajas 2016 [21]	High-Grade Glioma (*n* = 4)	^18^F-FMISO PET/MRI	Assessing Treatment Response	Histopathology
Barajas 2022 [22]	Glioblastoma (*n* = 6)	^18^F-FMISO PET/MRI	Assessing Treatment Response	Histopathology
Bekaert 2017 [23]	Glioma (*n* = 33)	^18^F-FMISO PET/CT	Correlation with Immunohistochemical Markers; Grading Gliomas; Survival Prognostication	Histopathology
Beppu 2014 [24]	Glioblastoma (*n* = 12)	^18^F-FRP170 PET/CT	Correlation with Immunohistochemical Markers	Histopathology
Beppu 2015 [25]	Glioblastoma (*n* = 13)	^18^F-FRP170 PET/CT	Correlation with Immunohistochemical Markers	Histopathology
Bruehlmeier 2004 [26]	Benign and Malignant Brain Tumours (*n* = 11)	^18^F-FMISO PET	Differentiating Gliomas from Other Brain Tumours; Grading Gliomas	Histopathology
Chakhoyan 2017 [27]	Glioma (*n* = 13)	^18^F-FMISO PET/CT	Grading Gliomas	Histopathology
Cher 2006 [28]	Primary and Metastatic Brain Tumours (*n* = 16)	Co-registered ^18^F-FMISO PET and MRI	Differentiating Gliomas from Other Brain Tumours; Grading Gliomas; Correlation with Immunohistochemical Markers; Survival Prognostication	Histopathology
Chvetsov 2024 [29]	Glioblastoma (*n* = 22)	^18^F-FMISO PET or PET/CT	Survival Prognostication	Histopathology
Gerstner 2016 [30]	Glioblastoma (*n* = 42)	^18^F-FMISO PET/CT	Survival Prognostication; Assessing Treatment Response	Histopathology
Hino-Shishikura 2014 [31]	Glioma; Primary CNS Lymphoma (*n* = 34)	^62^Cu-ATSM PET/CT	Differentiating Gliomas from Other Brain Tumours; Grading Gliomas	Histopathology
Hirata 2012 [32]	Glioma (*n* = 23)	^18^F-FMISO PET or PET/CT	Grading Gliomas	Histopathology
Hu 2020 [33]	Glioma (*n* = 25)	^18^F-FETNIM PET/CT	Grading Gliomas; Survival Prognostication; Correlation with Immunohistochemical Markers	Histopathology
Huang 2021 [34]	Glioblastoma (*n* = 33)	^18^F-FMISO PET or PET/CT	Survival Prognostication	Histopathology; RANO for progression assessment
Kanoto 2018 [35]	Glioma (*n* = 41)	^18^F-FMISO PET/CT	Grading Gliomas	Histopathology
Kawai 2011 [36]	Glioblastoma (*n* = 10)	^18^F-FMISO PET	Correlation with Immunohistochemical Markers	Histopathology
Kawai 2014 [37]	High-Grade Glioma (*n* = 32)	^18^F-FMISO PET or PET/CT	Grading Gliomas; Survival Prognostication; Correlation with Immunohistochemical Markers	Histopathology
Kobayashi 2020 [38]	Primary and Metastatic Brain Tumours (*n* = 23)	^18^F-FMISO PET/CT	Differentiating Gliomas from Other Entities; Grading Gliomas	Histopathology
Leimgruber 2019 [39]	Glioblastoma (*n* = 10)	^18^F-FMISO PET	Survival Prognostication; Assessing Treatment Responses	Histopathology
Mapelli 2021 [40]	High-Grade Glioma (*n* = 17)	^18^F-FAZA PET/CT	Correlation with Immunohistochemical Markers	Histopathology
Miyake 2021 [41]	Glioma (*n* = 113)	^18^F-FMISO PET/CT	Grading Gliomas; Predicting Gene Mutation Status	Histopathology; Molecular Analyses
Muzi 2015 [42]	Glioma (*n* = 38)	^18^F-FMISO PET or PET/CT	Survival Prognostication	Histopathology
Muzi 2020 [43]	High-Grade Glioma (*n* = 72)	^18^F-FMISO PET, PET/CT, or PET/MRI	Survival Prognostication	Histopathology
Shibahara 2010 [44]	Glioma (*n* = 8)	^18^F-FRP170 PET/CT	Grading Gliomas; Correlation with Immunohistochemical Markers	Histopathology
Shimizu 2020 [45]	Primary Brain Tumours (*n* = 15)	^18^F-FMISO PET or PET/CT	Differentiating Gliomas from Other Brain Tumours; Grading Gliomas	Histopathology
Spence 2008 [46]	Glioblastoma (*n* = 22)	^18^F-FMISO PET	Survival Prognostication; Correlation with Immunohistochemical Markers	Histopathology
Suzuki 2021 [47]	High-Grade Glioma (*n* = 87)	^18^F-FMISO PET or PET/CT	Predicting Gene Mutation Status	Histopathology
Suzuki 2023 [48]	Glioblastoma (*n* = 7)	^18^F-FMISO PET/CT	Correlation with Immunohistochemical Markers; Assessing Treatment Response	Histopathology; Molecular Analyses
Swanson 2009 [49]	Glioblastoma (*n* = 24)	^18^F-FMISO PET	Survival Prognostication; Assessing Treatment Response	Histopathology
Tateishi 2013 [50]	Glioma (*n* = 22)	^62^Cu-ATSM PET/CT	Correlation with Immunohistochemical Markers; Grading Gliomas	Histopathology
Tateishi 2014 [51]	Glioma (*n* = 23)	^62^Cu-ATSM PET/CT	Grading Gliomas	Histopathology
Toriihara 2018 [52]	Gliomas (*n* = 56)	^62^Cu-ATSM PET/CT	Survival Prognostication	Histopathology
Toyonaga 2016 [53]	Primary and Metastatic Brain Tumours (*n* = 59)	^18^F-FMISO PET or PET/CT	Differentiating Gliomas from Other Brain Tumours	Histopathology
Toyonaga 2016b [54]	Glioblastoma (*n* = 32)	^18^F-FMISO PET or PET/CT	Survival Prognostication	Histopathology
Uchinomura 2022 [55]	Glioblastoma and Primary CNS Lymphoma (*n* = 75)	^18^F-FMISO PET/CT	Differentiating Gliomas from Other Brain Tumours	Histopathology
Wang 2023 [56]	High-Grade Glioma (*n* = 35)	^18^F-FMISO PET/CT	Predicting Gene Mutation Status	Histopathology
Yamaguchi 2016 [57]	Glioma (*n* = 18)	^18^F-FMISO PET/CT	Survival Prognostication; Assessing Treatment Response	Histopathology

Note.--−^62^Cu-ATSM = ^62^Cu-diacetyl-bis (N4-methylthiosemicarbazone); ^18^F-FAZA = ^18^F-fluoroazomycin arabinoside; ^18^F-FETNIM = ^18^F-fluoroerythronitroimidazole; ^18^F-FMISO = ^18^F-fluoromisonidazole; ^18^F-FRP170 = 1-(2-fluoro-1-[hydroxymethyl]ethoxy)methyl-2-nitroimidazole; CNS = Central Nervous System; CT = Computed Tomography; MRI = Magnetic Resonance Imaging RANO = Response Assessment in Neuro-Oncology; PET = Positron Emission Tomography.

## Data Availability

No new data were created or analyzed in this study. Data sharing is not applicable to this article.

## Supplementary Materials

The following supporting information can be downloaded at: https://www.mdpi.com/article/10.3390/cancers18121898/s1, Figure S1. Summary of quality assessment data for all included studies which were included in this review. (A) Traffic light plot of QUADAS-2 assessment for all included diagnostic accuracy studies. (B) Traffic light plot of QUIPS assessment for all included prognostic studies. (C) Unweighted summary bar plot illustrating the distribution of risk of bias and applicability concerns for all diagnostic accuracy studies, according to QUADAS-2. (D) Unweighted summary bar plot illustrating the distribution of risk of bias for all prognostic studies, according to QUIPS. Plots created using RobVIS; Figure S2**. **Overview of meta-analysis results for the diagnostic value of hypoxia PET for glioma (A) Forest plots for the pooled sensitivity and specificity calculation for differentiating glioblastoma from all other CNS lesions. Horizontal lines represent 95% confidence intervals of the individual studies. The dashed red line is the line of summary points across the studies (B) Likelihood ratio scattergram for glioblastoma assessment (C) Hierarchical summary receiver operating characteristic (SROC) curve for overall response assessment. The “observed data” points show accuracy for each study, and the “summary operating point” represents the pooled accuracy; Figure S3**. **Funnel plot with superimposed regression line to investigate publication bias in the dataset for the diagnostic value of ^18^F-FMISO PET in distinguishing glioblastoma from lower-grade gliomas; Table S1. Detailed information on imaging protocols, patient populations, and reference standards for all included studies; Table S2. Summary of studies assessing the performance of hypoxia PET for predicting immunohistochemical markers. File S1: PRISMA-DTA Checklist.

## Abbreviations

The following abbreviations are used in this manuscript:

ATSM	diacetyl-bis (N4-methylthiosemicarbazone)
CA-IX	Carbonic Anhydrase 9
CI	Confidence Interval
CNS	Central Nervous System
CT	Computed Tomography
FAZA	Fluoroazomycin Arabinoside
FDG	Fluorodeoxyglucose
FET	Fluoroethyl-l-tyrosine
FETNIM	Fluoroerythronitroimidazole
FMISO	Fluoromisonidazole
FN	False Negative
FP	False Positive
FRP170	1-(2-fluoro-1-[hydroxymethyl]ethoxy)methyl-2-nitroimidazole
GBM	Glioblastoma
HIF-1α	Hypoxia-Inducible Factor 1-Alpha
HV	Hypoxic Volume
LR	Likelihood Ratio
MET	Methionine
MRI	Magnetic Resonance Imaging
OS	Overall Survival
PCR	Polymerase Chain Reaction
PET	Positron Emission Tomography
PFS	Progression-Free Survival
SUV	Standardized Uptake Value
TP	True Positive
TN	True Negative
TTP	Time To Progression
VEGF	Vascular Endothelial Growth Factor
WHO	World Health Organization
