# Supplementary material for: Diagnostic and Prognostic Value of Hypoxia PET in Glioma: A Systematic Review and Meta-Analysis
Source: Cancers (Basel). 2026 Jun 10;18(12):1898. doi: 10.3390/cancers18121898 (PMC13297157; doi:10.3390/cancers18121898)
Supplement: Supplementary file 1 [file cancers-18-01898-s001.zip › Supplemental Figures.pdf]

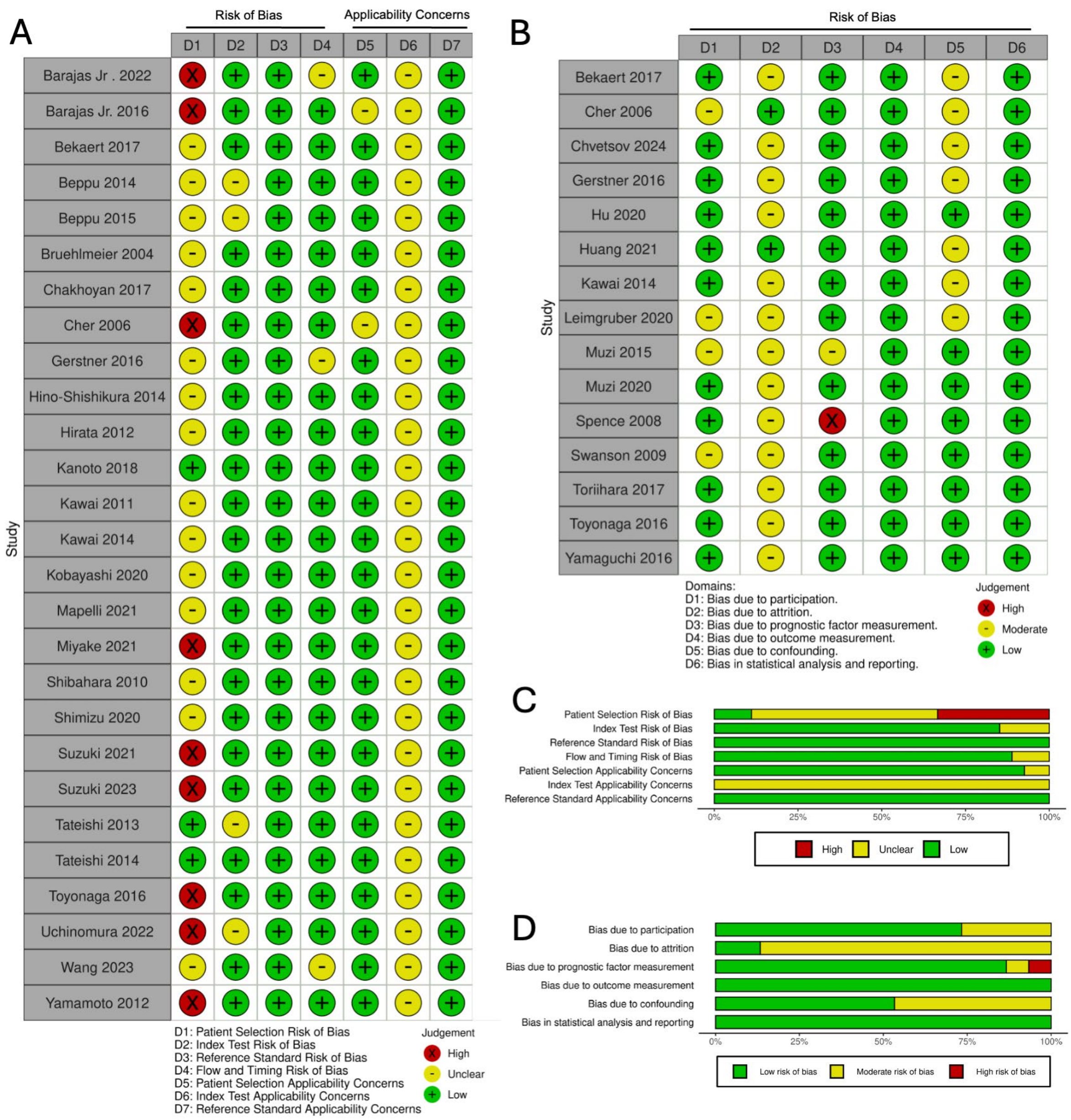

Figure S1. Summary of quality assessment data for all included studies which were included in this review. (**A**) Traffic light plot of QUADAS-2 assessment for all included diagnostic accuracy studies. (**B**) Traffic light plot of QUIPS assessment for all included prognostic studies. (**C**) Unweighted summary bar plot illustrating the distribution of risk of bias and applicability concerns for all diagnostic accuracy studies, according to QUADAS-2. (**D**) Unweighted summary bar plot illustrating the distribution of risk of bias for all prognostic studies, according to QUIPS. Plots created using RobVIS.

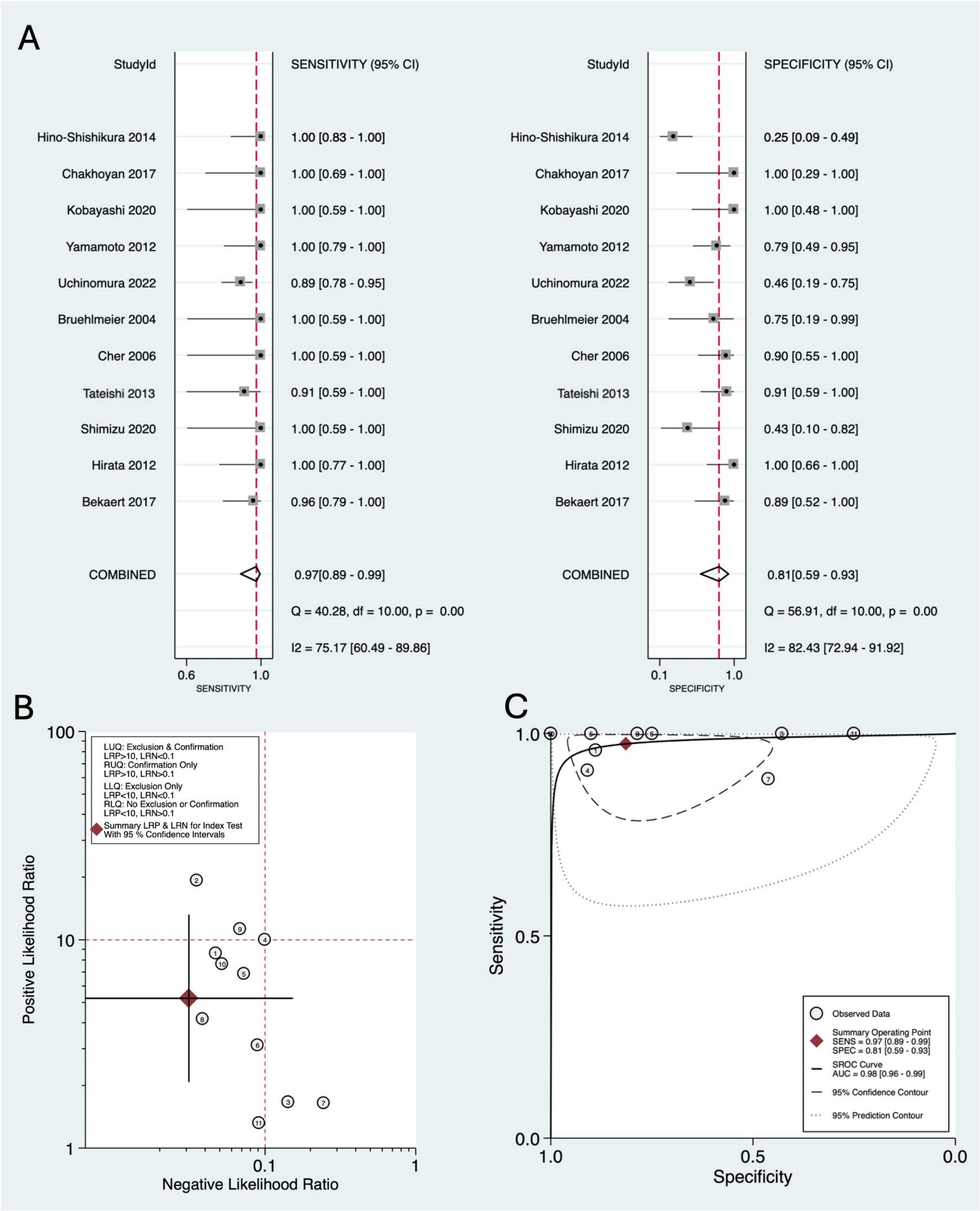

Figure S2. Overview of meta-analysis results for the diagnostic value of hypoxia PET for glioma (**A**) Forest plots for the pooled sensitivity and specificity calculation for differentiating glioblastoma from all other CNS lesions. Horizontal lines represent 95% confidence intervals of the individual studies. The dashed red line is the line of summary points across the studies (**B**) Likelihood ratio scattergram for glioblastoma assessment (**C**) Hierarchical summary receiver operating characteristic (SROC) curve for overall response assessment. The "observed data" points show accuracy for each study, and the "summary operating point" represents the pooled accuracy.

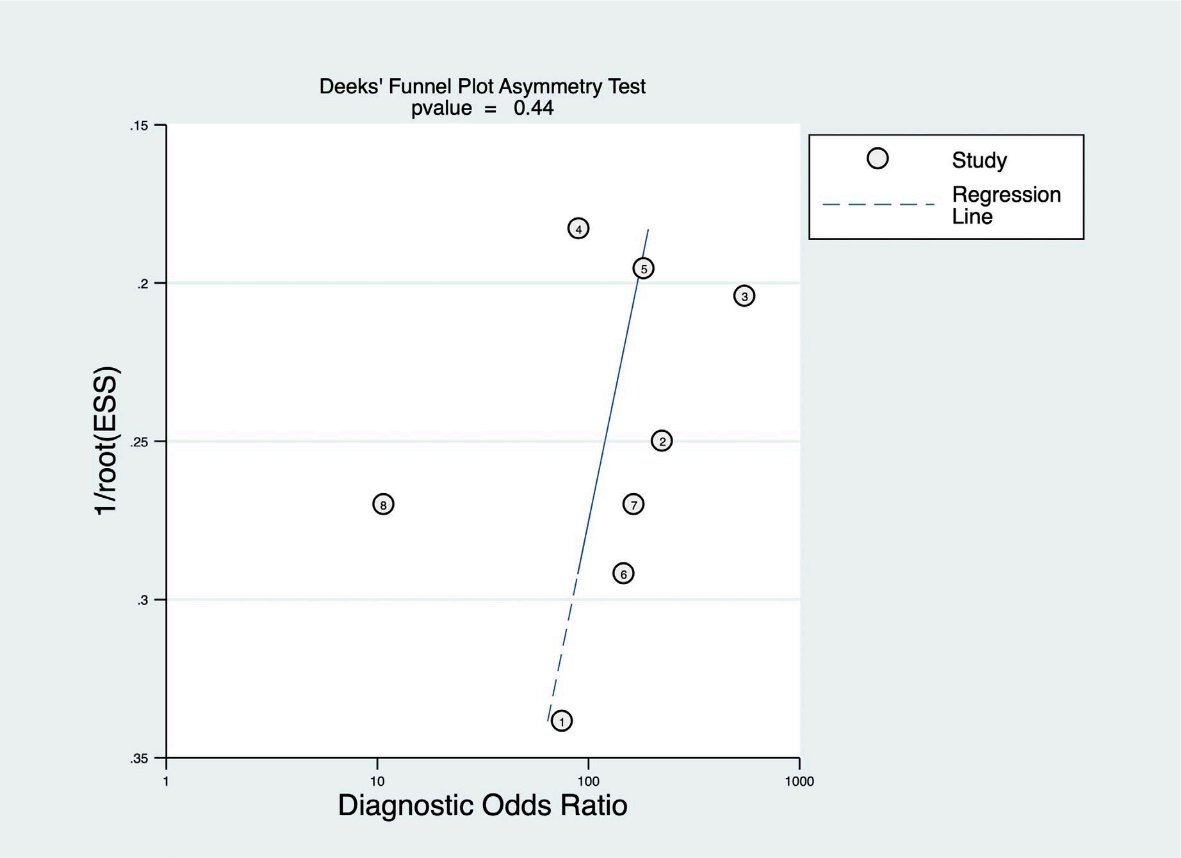

Figure S3. Funnel plot with superimposed regression line to investigate publication bias in the dataset for the diagnostic value of ^{18}F-FMISO PET in distinguishing glioblastoma from lower-grade gliomas.
