# Supplementary material for: Diagnostic and Prognostic Value of Hypoxia PET in Glioma: A Systematic Review and Meta-Analysis
Source: Cancers (Basel). 2026 Jun 10;18(12):1898. doi: 10.3390/cancers18121898 (PMC13297157; doi:10.3390/cancers18121898)
Supplement: Supplementary file 1 [file cancers-18-01898-s001.zip › Supplemental Table S1.pdf]

Supplemental Table S1. Detailed information on imaging protocols, patient populations, and reference standards for all included studies.

First Author and Year	Diagnoses and No. of Patients	Male: Female (% Male)	Age	Tracer and Imaging Modality	Outcome(s) Evaluated	PET, PET/CT or PET/MR Device	PET Acquisition Details
Barajas 2016 [21]	High-Grade Glioma (n = 4)	3:1 (75%)	Mean = 55 years	^{18}F-FMISO PET/MRI	Assessing Treatment Response	3T investigational GE PET/MRI scanner	40-minute acquisition carried out 90 minutes after 259 MBq ^{18}F-FMISO administration
Barajas 2022 [22]	Glioblastoma (n = 6)	5:1 (83%)	Mean = 56.5 years	^{18}F-FMISO PET/MRI	Assessing Treatment Response	Not Listed	Acquisition carried out 90 minutes after 3.7 MBq/kg ^{18}F-FMISO administration
Bekaert 2017 [23]	Glioma (n = 33)	24:9 (73%)	Mean = 58 years	^{18}F-FMISO PET/CT	Correlation with Immunohistochemical Markers; Grading Gliomas; Survival Prognostication	GE Healthcare Discovery RX VCT HD	20-minute acquisition carried out 120 minutes after 5 MBq/kg ^{18}F-FMISO administration
Beppu 2014 [24]	Glioblastoma (n = 12)	10:2 (83%)	Mean = 63 years	^{18}F-FRP170 PET/CT	Correlation with Immunohistochemical Markers	Eminence Sophia SET3000 GCT/M	Acquisition carried out 60 minutes after 370 MBq ^{18}F-FRP170 administration
Beppu 2015 [25]	Glioblastoma (n = 13)	9:4 (69%)	Mean = 60 years	^{18}F-FRP170 PET/CT	Correlation with Immunohistochemical Markers	Eminence Sophia SET3000 GCT/M	Acquisition carried out 60 minutes after 370 MBq ^{18}F-FRP170 administration
Bruehlmeier 2004 [26]	Benign and Malignant Brain Tumours (n = 11)	8:3 (73%)	Mean = 49 years	^{18}F-FMISO PET	Differentiating Gliomas from Other Brain Tumours; Grading Gliomas	GE Advance	Dynamic PET acquisition over 90 minutes beginning at administration of ~291 MBq of ^{18}F-FMISO<br><br>Static 20-minute acquisition carried out 150–170 minutes after ^{18}F-FMISO injection

Chakhoyan 2017 [27]	Glioma (*n* = 13)	12:1 (92%)	Mean = 56 years	^{18}F-FMISO PET/CT	Grading Gliomas	GE Discovery VCT 64	20-minute acquisition carried out 120 minutes after ~5 MBq/kg ^{18}F-FMISO administration
Cher 2006 [28]	Primary and Metastatic Brain Tumours (*n* = 16)	10:6 (63%)	Mean = 49 years	Co-registered ^{18}F-FMISO PET and MRI	Differentiating Gliomas from Other Brain Tumours; Grading Gliomas; Correlation with Immunohistochemical Markers; Survival Prognostication	Siemens ECAT 951/31R	Acquisition carried out 120 minutes after 18.5 MBq/kg ^{18}F-FMISO administration
Chvetsov 2024 [29]	Glioblastoma (*n* = 22)	15:6 (68%)	Mean = 59 years	^{18}F-FMISO PET or PET/CT	Survival Prognostication	GE Advance, GE Discovery	20-minute acquisition carried out 110 minutes after ^{18}F-FMISO administration
Gerstner 2016 [30]	Glioblastoma (*n* = 42)	27:15 (64%)	Median = 59 years	^{18}F-FMISO PET/CT	Survival Prognostication; Assessing Treatment Response	Not Listed	20-minute acquisition carried out 110 minutes after 3.7 MBq/kg ^{18}F-FMISO administration
Hino-Shishikura 2014 [31]	Glioma; Primary CNS Lymphoma (*n* = 34)	18:16 (53%)	Mean = 56 years	^{62}Cu-ATSM PET/CT	Differentiating Gliomas from Other Brain Tumours; Grading Gliomas	Aquiduo PCA-7000B	Dynamic scans taken for 30 mins after the injection of 740 MBq of ^{62}Cu-ATSM<br>Static PET/CT images reconstructed with the last 10 minutes of the acquisition (20–30 minutes after administration)
Hirata 2012 [32]	Glioma (*n* = 23)	10:13 (43%)	Mean = 57.0 years	^{18}F-FMISO PET or PET/CT	Grading Gliomas	Siemens/CTI ECAT EXACT HR+, Siemens/CTI Biograph 64	Acquisition carried out 4 hours after 400 MBq ^{18}F-FMISO administration

Hu 2020 [33]	Glioma (*n* = 25)	16:9 (64%)	68% of patients were ≤ 40 years	^{18}F-FETNIM PET/CT	Grading Gliomas; Survival Prognostication; Correlation with Immunohistochemical Markers	GE Healthcare Discovery LS	Acquisition carried out 120 minutes after 3.7 MBq/kg ^{18}F-FETNIM administration
Huang 2021 [34]	Glioblastoma (*n* = 33)	22:11 (67%)	Mean = 46 years	^{18}F-FMISO PET or PET/CT	Survival Prognostication	Siemens/CTI EXACT HR; Siemens Biograph40 mCT	20-minute acquisition carried out approximately 120 minutes after ^{18}F-FMISO administration
Kanoto 2018 [35]	Glioma (*n* = 41)	21:20 (51%)	Mean = 57 years	^{18}F-FMISO PET/CT	Grading Gliomas	Siemens/CTI Biograph mCT	Acquisition carried out 4 hours after 400 MBq ^{18}F-FMISO administration
Kawai 2011 [36]	Glioblastoma (*n* = 10)	7:3 (70%)	Mean = 58 years	^{18}F-FMISO PET	Correlation with Immunohistochemical Markers	Siemens/CTI ECAT EXACT HR+	10-minute acquisition carried out 120–140 minutes after ~380 MBq ^{18}F-FMISO administration
Kawai 2014 [37]	High-Grade Glioma (*n* = 32)	23:25 (48%)	Mean = 51 years	^{18}F-FMISO PET or PET/CT	Grading Gliomas; Survival Prognostication; Correlation with Immunohistochemical Markers	Siemens/CTI ECAT EXACT HR+; Siemens/CTI Biograph mCT	10-minute acquisition carried out 120 minutes after ~270 MBq ^{18}F-FMISO administration
Kobayashi 2020 [38]	Primary and Metastatic Brain Tumours (*n* = 23)	10:13 (43%)	Median = 61	^{18}F-FMISO PET/CT	Differentiating Gliomas from Other Entities; Grading Gliomas	Hitachi Gemini GXL 16	20-minute acquisitions carried out 120 minutes and 240 minutes after ~395 MBq ^{18}F-FMISO administration
Leimgruber 2019 [39]	Glioblastoma (*n* = 10)	5:5 (50%)	Mean = 61 years	^{18}F-FMISO PET	Survival Prognostication	Philips PET Scanner (not named)	Acquisition carried out 2 hours after 1.85 MBq/kg ^{18}F-FMISO administration
Mapelli 2021 [40]	High-Grade Glioma (*n* = 17)	12:5 (71%)	Mean = 64.6 years	^{18}F-FAZA PET/CT	Correlation with Immunohistochemical Markers	GE Discovery 690	Acquisition carried out 120 minutes after 372±17 MBq ^{18}F-FAZA administration

Miyake 2021 [41]	Glioma (*n* = 113)	53:60 (47%)	Median = 56.7	^{18}F-FMISO PET/CT	Grading Gliomas; Predicting Gene Mutation Status	Siemens Biograph mCT	Dose and timing not mentioned
Muzi 2015 [42]	Glioma (*n* = 38)	Not Reported	Not Reported	^{18}F-FMISO PET or PET/CT	Survival Prognostication	GE Advance, GE Healthcare	20-minute acquisition carried out 90–140 minutes after 148–370 MBq ^{18}F-FMISO administration
Muzi 2020 [43]	High-Grade Glioma (*n* = 72)	Cohort 1: 21:9 (70%)<br><br>Cohort 2: 27:15 (64%)	Cohort 1: Median = 58 years<br><br>Cohort 2: Median = 60 years	^{18}F-FMISO PET/CT or PET/MRI	Survival Prognostication	GE Advance, GE Discovery LS, GE Discovery RX, GE Discovery STE, Prototype Siemens Brain MRI/PET, Siemens ECAT HR+, Siemens Biograph 64, Siemens Biograph 40	20-minute acquisition carried out ~110 minutes after ^{18}F-FMISO administration
Shibahara 2010 [44]	Glioma (*n* = 8)	5:3 (63%)	Mean = 49 years	^{18}F-FRP170 PET/CT	Grading Gliomas; Correlation with Immunohistochemical Markers	Siemens ECAT EXACT HR+	Acquisition carried out 120 minutes after 370 MBq ^{18}F- FRP170 administration
Shimizu 2020 [45]	Primary Brain Tumours (*n* = 15)	8:7 (53%)	Mean = 54 years	^{18}F-FMISO PET or PET/CT	Differentiating Gliomas from Other Brain Tumours; Grading Gliomas	Siemens Biograph 64, Siemens ECAT EXACT HR+, or Hitachi Gemini TF64 TOF	10-minute acquisition carried out 4 hours after 5 MBq/kg ^{18}F-FMISO administration
Spence 2008 [46]	Glioblastoma (*n* = 22)	13:9 (59%)	Median = 56 years	^{18}F-FMISO PET	Survival Prognostication; Correlation with Immunohistochemical Markers	GE Advance	20-minute acquisition carried out 120 minutes after 3.7 MBq/kg ^{18}F-FMISO administration
Suzuki 2021 [47]	High-Grade Glioma (*n* = 87)	45:42 (52%)	Median = 64 years	^{18}F-FMISO PET or PET/CT	Predicting Gene Mutation Status	Siemens ECAT EXACT HR+, Siemens Biograph mCT	10-minute acquisition carried out 120 minutes after ~262 MBq ^{18}F-FMISO administration

Suzuki 2023 [48]	Glioblastoma (*n* = 7)	5:2 (71%)	Mean = 64 years	^{18}F-FMISO PET/CT	Correlation with Immunohistochemical Markers; Assessing Treatment Response; Survival Prognostication	Siemens Biograph mCT	10-minute acquisition carried out 120 minutes after ^{18}F-FMISO administration
Swanson 2009 [49]	Glioblastoma (*n* = 24)	16:8 (67%)	Mean = 55 years	^{18}F-FMISO PET	Survival Prognostication; Assessing Treatment Response	GE Advance	20-minute acquisition carried out 120 to 140 minutes after 3.7 MBq/kg ^{18}F-FMISO administration
Tateishi 2013 [50]	Glioma (*n* = 22)	6:16 (27%)	Mean = 56.4 years	^{62}Cu-ATSM PET/CT	Survival Prognostication; Correlation with Immunohistochemical Markers; Grading Gliomas	Toshiba Aquiduo PCA-7000B	Dynamic scans were taken for 40 minutes after ~740 MBq ^{62}Cu-ATSM administration<br>Static images were reconstructed from the last 10 minutes (minutes 30–40 after administration)
Tateishi 2014 [51]	Glioma (*n* = 23)	10:13 (43%)	Mean = 54 years	^{62}Cu-ATSM PET/CT	Grading Gliomas	Toshiba Aquiduo PCA-7000B	Dynamic scans were taken for 40 minutes after ~740 MBq ^{62}Cu-ATSM administration<br>Static images were reconstructed from the last 10 minutes (minutes 30–40 after administration)
Toriihara 2018 [52]	Gliomas (*n* = 56)	28:28 (50%)	Mean = 56 years	^{62}Cu-ATSM PET/CT	Survival Prognostication	Not mentioned	Dynamic scans taken for 30 mins after the injection of ~482 MBq of ^{62}Cu-ATSM<br>Static images reconstructed from the last 10 minutes (minutes 20–30 after administration)
Toyonaga 2016 [53]	Primary and Metastatic Brain	33:26 (56%)	Range: 30–85	^{18}F-FMISO PET or PET/CT	Differentiating Gliomas from Other Brain Tumours	Siemens ECAT HR+ , Siemens Biograph 64, and Gemini TF64 TOF	10-minute acquisition carried out 4 hours after ~400 MBq ^{18}F-FMISO administration

	Tumours (*n* = 59)						
Toyonaga 2016 [54]	Glioblastoma (*n* = 32)	17:15 (53%)	Mean = 63.5 years	^{18}F-FMISO PET or PET/CT	Survival Prognostication	Siemens ECAT EXACT HR+, Siemens Biograph 64, Hitachi Gemini TF64	Acquisition carried out 60 minutes after 4.5 MBq/kg ^{18}F-FMISO administration
Uchinomura 2022 [55]	Glioblastoma and Primary CNS Lymphoma (*n* = 75)	41:34 (55%)	Mean = 67 years	^{18}F-FMISO PET/CT	Grading Gliomas	Siemens Biograph mCT	15-minutes acquisition carried out 120 minutes after 3.7 MBq/kg ^{18}F-FMISO administration
Wang 2023 [56]	High-Grade Glioma (*n* = 35)	20:15 (57%)	Mean = 50	^{18}F-FMISO PET/CT	Predicting Gene Mutation Status	GE Healthcare Discovery IQ	15-minute acquisition carried out 4 hours after 350–550 MBq ^{18}F-FMISO administration
Yamaguchi 2016 [57]	Glioma (*n* = 18)	8:10 (44%)	Mean = 60 years	^{18}F-FMISO PET/CT	Survival Prognostication	Siemens Biograph 64<br><br>Hitachi Gemini TF64 TOF	Acquisition carried out 4 hours after 400 MBq ^{18}F-FMISO administration
Yamamoto 2012 [58]	Glioma (*n* = 30)	12:18 (40%)	Mean = 48 years	^{18}F-FMISO PET/CT	Grading Gliomas	Siemens Biograph mCT	Acquisition carried out 120 minutes after 3.7 MBq/kg ^{18}F-FMISO administration

Note.---^{62}Cu-ATSM = ^{62}Cu-diacetyl-bis (N4-methylthiosemicarbazone); ^{18}F-FAZA = ^{18}F-fluoroazomycin arabinoside; ^{18}F-FETNIM = ^{18}F-fluoroerythronitroimidazole; ^{18}F-FMISO = ^{18}F-fluoromisonidazole; ^{18}F-FRP170 = 1-(2-fluoro-1-[hydroxymethyl]ethoxy)methyl-2-nitroimidazole; CNS = Central Nervous System; CT = Computed Tomography; MRI = Magnetic Resonance Imaging; PET = Positron Emission Tomography
