# Supplementary material for: Diagnostic and Prognostic Value of Hypoxia PET in Glioma: A Systematic Review and Meta-Analysis
Source: Cancers (Basel). 2026 Jun 10;18(12):1898. doi: 10.3390/cancers18121898 (PMC13297157; doi:10.3390/cancers18121898)
Supplement: Supplementary file 1 [file cancers-18-01898-s001.zip › Supplemental Table S2.pdf]

Supplemental Table S2. Summary of studies assessing the performance of hypoxia PET for predicting immunohistochemical markers

Paper	Tracer	No. of Patients	Included Diagnoses	Interpretation Parameters	Histological Markers	Performance
Bekaert 2017 [23]	^{18}F-FMISO	33	Grade II/III/IV Glioma	SUVmax, Hypoxic Volume (HV)	CA-IX	Significantly Positively Correlated with both SUVmax (Spearman rho = 0.636, *p* < 0.001) and HV (Spearman rho = 0.616, *p* = 0.001)
					HIF-1a	Significantly Positively Correlated with both SUVmax (Spearman rho = 0.459, *p* = 0.014) and HV (Spearman rho = 0.521, *p* = 0.004)
					VEGF	Significantly Positively Correlated with both SUVmax (Spearman rho = 0.498, *p* = 0.008) and HV (Spearman rho = 0.486, *p* = 0.010)
					Ang2	Significantly Positively Correlated with both SUVmax (Spearman rho = 0.515, *p* = 0.006) and HV (Spearman rho = 0.475, *p* = 0.017)
Beppu 2014 [24]	^{18}F-FRP170	12	Glioblastoma	High-Uptake Areas vs. Low-Uptake Areas	HIF-1a	High-Uptake Areas: all nuclei positive, 3/6 cytoplasms positive for HIF-1a<br><br>Low-uptake areas: few cells positive in 1/6, cytoplasm only in 3/6, and cytoplasm and nuclei positive in 2/6
Beppu 2015 [25]	^{18}F-FRP170	13	Glioblastoma	FRP170 high-uptake vs low-uptake areas	HIF-1a	Significant difference (*p* < 0.01) in % positivity between FRP-170 high- and low-uptake areas
					Ki-67	No significant difference between FRP-170 high- and low-uptake areas
Cher 2006 [28]	^{18}F-FMISO	14	Grade I/II/III/IV Glioma	Visual Uptake	HIF-1a	Not significantly correlated with ^{18}F-FMISO uptake
					Glucose Transporter 3	Not significantly correlated with ^{18}F-FMISO uptake
					Tumour Vascularity	Not significantly correlated with ^{18}F-FMISO uptake
					Ki-67	Significantly correlated with ^{18}F-FMISO uptake
					VEGF	Not significantly correlated with ^{18}F-FMISO uptake
					VEGF-R1	Significantly correlated with ^{18}F-FMISO uptake
Hu 2020 [33]	^{18}F-FETNIM	25	High-Grade (Grade	SUVmax	HIF-1a	Significantly correlated with SUVmax (r = 0.820, *p* < 0.001)
					Ki-67	Significantly correlated with SUVmax (r = 0.747, *p* < 0.001)
					VEGF	Significantly correlated with SUVmax (r = 0.606, *p* < 0.001)

			III/IV) Glioma		MMP-9	Significantly correlated with SUVmax (r = 0.727, *p* < 0.001)
Kawai 2011 [36]	^{18}F-FMISO	10	Glioblastoma	HV	Ki67	Not significantly correlated with ^{18}F-FMISO uptake (*p* > 0.05)
Kawai 2014 [37]	^{18}F-FMISO	32	High-Grade (Grade III/IV) Glioma	SUVmax	HIF-1a	Semiquantitative immunoreactivity score (0-12) did not significantly differ between grade III and IV gliomas; immunoreactivity did not correlate significantly with SUVmax
					VEGF	Semiquantitative immunoreactivity score (0-12) did not significantly differ between grade III and IV gliomas; immunoreactivity was weakly correlated with SUVmax
Mapelli 2021 [40]	^{18}F-FAZA	17	High-Grade (Grade III/IV) Glioma	SUVmax, SUVmean, FAZA tumour volume (FTV), hypoxic volume	CA-IX	In surgical samples (*n* = 7), Significantly correlated with SUVmax (*p* = 0.0002), SUVmean40 (*p* = 0.0058), SUVmean50 (*p* = 0.009), SUVmean60 (*p* = 0.0153), FTV–40–50–60 (*p* = 0.0424) and HV1.2-1.3-1.4 (*p* = 0.0058)
					Tumour Vascularity	In the biopsy samples (*n* = 10), significantly inversely correlated with SUVmax (*p* = 0.0094), SUVmean40 (*p* = 0.0107),<br>SUVmean50 (*p* = 0.0094) and SUVmean60 (*p* = 0.0154)
Shibahara 2010 [44]	^{18}F-FRP170	8	Grade II/III/IV Glioma	SUVmax	HIF-1a	No quantitative evaluation of performance of PET parameters in correlating with HIF-1a expression, but it stained strongly in the 3 glioblastomas that were FRP-170 positive, and variable/moderate staining for the remainder of the tumours (lower-grade gliomas)
Spence 2008 [46]	^{18}F-FMISO	22	Glioblastoma	HV, T/Bmax	HIF-1a	No significant correlation with HV or T/Bmax
					Ki-67	No significant correlation with HV or T/Bmax
					VEGF	No significant correlation with HV or T/Bmax
					p53	No significant correlation with HV or T/Bmax
Suzuki 2023 [48]	^{18}F-FMISO	7	Glioblastoma	TBRmax	CA-IX	Correlated significantly with ^{18}F-FMISO TBRmax (r = 0.90, *p* = 0.006)
					PD-L1	Correlated significantly with ^{18}F-FMISO TBRmax (r = 0.88, *p* = 0.009)
Tateishi 2013 [50]	^{62}Cu-ATSM	22	Grade II/III/IV Glioma	T/B ratio > 1.8	HIF-1a	92.3% sensitivity and 88.9% specificity

Toyonaga 2016 [53]	^{18}F-FMISO	59	Primary and Metastatic Brain Tumours (including grade I/II/III/IV gliomas, PCNSL, metastatic brain tumours, hemangioblastomas, meningioma, craniopharyngioma)	Visual Assessment; T/N ratio > 1.67	Predicting Pathological Necrosis	T/N ratio > 1.67 was 96.7% sensitive and 93.1% specific for histological necrosis

Note.---^{62}Cu-ATSM = ^{62}Cu-diacetyl-bis (N4-methylthiosemicarbazone); ^{18}F-FAZA = ^{18}F-fluoroazomycin arabinoside; ^{18}F-FETNIM = ^{18}F-fluoroerythronitroimidazole; ^{18}F-FMISO = ^{18}F-fluoromisonidazole; ^{18}F-FRP170 = 1-(2-fluoro-1-[hydroxymethyl]ethoxy)methyl-2-nitroimidazole; Ang2 = Angiopoietin-2; CA-IX = Carbonic Anhydrase IX; CNS = Central Nervous System; CT = Computed Tomography; FTV = FAZA Tumour Volume; HIF-1a = Hypoxia-Inducible Factor 1 alpha; HV = hypoxic volume; Ki-67 = Kiel 67; MMP-9 = Matrix Metallopeptidase 9; MRI = Magnetic Resonance Imaging; PD-L1 = programmed death-ligand 1; PET = Positron Emission Tomography; SUVmax = maximum standardized uptake value; SUVmean = mean standardized uptake value; T/B = Tumour-to-Background ratio; T/Bmax = Tumour-to-Background SUVmax; TBRmax = Tumour-to-Blood SUVmax; VEGF = Vascular Endothelial Growth Factor; VEGF-R1 = Vascular Endothelial Growth Factor-Receptor 1
